# Downregulation of GLYR1 contributes to microsatellite instability colorectal cancer by targeting p21 via the p38MAPK and PI3K/AKT pathways

**DOI:** 10.1186/s13046-020-01578-y

**Published:** 2020-05-05

**Authors:** Zhiyan Hu, Ting Long, Yidan Ma, Jiaxian Zhu, Lingfang Gao, Yan Zhong, Xia Wang, Xiaoyan Wang, Zuguo Li

**Affiliations:** 1 Department of Pathology, Shenzhen Hospital of Southern Medical University, Shenzhen, China; 2 Department of Pathology, Nanfang Hospital, Southern Medical University, Guangzhou, China; 3 Department of Pathology, School of Basic Medical Sciences, Southern Medical University, Guangzhou, China; 4 Guangdong Provincial Key Laboratory of Molecular tumor Pathology, Guangzhou, China

**Keywords:** GLYR1, Microsatellite instability colorectal cancer, MLH1, p38MAPK, PI3K/Akt, p21

## Abstract

**Background:**

GLYR1 has a high mutation frequency in microsatellite instability colorectal cancer (MSI CRC) and is presumed to be a novel tumor suppressor. However, the role of GLYR1 in tumors has never been studied. In particular, the downregulation of GLYR1 in MSI CRC is worthy of further investigation.

**Methods:**

Western blot and immunohistochemistry analyses were used to detect GLYR1 protein expression in CRC tissues and cell lines, and the clinical significance of GLYR1 was also analyzed. The relationship between GLYR1 and MLH1 was validated by immunofluorescence, immunoprecipitation and bioinformatics analyses. Western blotting, qRT-PCR, CCK-8 assays, colony formation assays, flow cytometry and Hoechst 33258 staining assays were used to assess the effect of GLYR1 on the cell cycle progression, proliferation, differentiation and apoptosis of CRC cells in vitro. The related mechanisms were initially investigated by Western blotting.

**Results:**

GLYR1 was significantly downregulated in MSI CRC and its expression was negatively correlated with tumor size and positively correlated with tumor differentiation in CRC patients. In addition, GLYR1 interacted with MLH1 to regulate its nuclear import and expression. Moreover, downregulation of GLYR1 accelerated G1/S phase transition, promoted proliferation and inhibited differentiation of SW480 and SW620 cells in vitro. Furthermore, downregulation of GLYR1 decreased the sensitivity to 5-fluorouracil (5-FU) by inhibiting the mitochondrial apoptosis pathway in CRC cells. Inhibition of the p38 mitogen-activated protein kinase (p38MAPK) and activation of the phosphatidyl 3-kinase/protein kinase B (PI3K/Akt) signaling pathways were involved in the mechanism by which GLYR1 downregulated p21.

**Conclusions:**

Ours is the first study to elucidate the role of GLYR1 in tumors and provide evidence for GLYR1 as a biological marker that reflects the degree of malignancy and sensitivity to 5-FU in MSI CRC.

## Background

Colorectal cancer (CRC) is a common malignant tumor of the digestive system in worldwide. Approximately 15–20% of CRCs are caused by microsatellite instability (MSI) [1], which usually results from mutation of the mismatch repair (MMR) system genes (MLH1, PMS2, MSH2, and MSH6) or more commonly because of promoter methylation of MLH1 gene [2]. Microsatellite instability colorectal cancer (MSI CRC) is a special subtype of CRC with good prognosis and insensitivity to 5-fluorouracil (5-FU) chemotherapy [2–4]. Therefore, a novel marker for diagnosis and guidance in the use of 5-FU chemotherapy of MSI CRC is of great scientific significance and urgently needed.

Glyoxylate reductase 1 homolog (GLYR1), also known as NP60 and N-PAC, was originally discovered as an oxidoreductase in plant research. This protein, which is encoded by a gene located on the 16p13.3 chromosome, contains three domains: PWWP, AT-hook and NAD binding regions. The role of GLYR1 in tumors was first reported in 2012 by Alhopuro et al., who found that GLYR1 had a mutation frequency of 51% in MSI CRC and presumed it to be a novel tumor suppressor [5].

Recently, GLYR1 overexpressoin was reported to induce p21 transcriptional activation and increase caspase activity via a p53-independent pathway [6]. p21, which is encoded by the CDKN1A gene, is an important member of cyclin-dependent kinase (CDK) inhibitors family that participates in the regulation of G1 to S phase transition by inhibiting the activity of the CDK2/cyclinE complex [7, 8]. In the cell cycle, one of the regulatory points (R) that determine cell differentiation or proliferation is located in the G1 phase [9, 10]. G1 phase regulation is closely related to cell differentiation, proliferation and various stress responses [11]. Studies on the regulatory mechanisms of p21 are focused mainly on p53-dependent or p53-independent pathways. Most of these studies have indicated that p21 is directly targeted by p53, although more recent studies have indicated that p21 regulates cell cycle arrest, cell proliferation, apoptosis and other functions via a p53-independent pathway [12–15]. Furthermore, the well-characterized p38 mitogen-activated protein kinase (p38MAPK) and phosphatidyl 3-kinase/protein kinase B (PI3K/Akt) signaling pathways have been shown to be involved in the regulation of p21 [16–18]. The p38MAPK signaling pathway, which is common in most cells, transduces cytoplasmic signals into the nucleus and activates downstream signaling pathways involved in processes such as cell metabolism, growth, proliferation, survival, gene transcription and protein synthesis [19, 20]. The PI3K/AKT signaling pathway, widely known as a classical anti-apoptotic pathway, is aberrantly activated in a variety of tumors and participates in the regulation of tumor growth and survival. Recent studies have shown that the PI3K/AKT signaling pathway is closely associated with resistance to 5-FU chemotherapy in CRC.

Although GLYR1 has been shown to specifically regulate p38MAPK14 signaling [21], the role of GLYR1 in CRC occurrence, development and therapy as well as the underlying mechanism remains to be determined. In particular, the downregulation of GLYR1 in MSI CRC is worthy of further investigation. In the present study, we demonstrated the expression and clinical significance of GLYR1 in CRC samples and further studied its relationship with expression of the mismatch repair gene MLH1. We then investigated the effects of GLYR1 downregulation on cell cycle progression, proliferation, differentiation and apoptosis in CRC cells in vitro. Subsequently, we further explored the mechanism by which GLYR1 downregulated p21.

## Materials and methods

### Clinical samples and cell lines

A total of 221 paraffin-embedded human CRC tissue sections used in this study were obtained from the Department of Pathology, Nanfang Hospital, Southern Medical University, China. In each case, a definite diagnosis of primary CRC was confirmed. This study was approved by the ethics committee of Nanfang Hospital. The CRC cell lines SW620, SW480, HT29, Caco-2, LS174T, HCT8, HCT116, DLD1, and LOVO and one normal human fetal colonic mucosa cell line FHC were purchased from the American Type Culture Collection (ATCC, USA) and preserved in the Department of Pathology, School of Basic Medical Sciences, Southern Medical University, Guangzhou, China. According to reports [22, 23], SW620, SW480, HT29 and Caco-2 were MSS CRC cell lines, while LS174T, HCT8, HCT116, DLD1 and LOVO were MSI CRC cell lines. Cells were cultured in RPMI 1640 medium (Gibco, USA) supplemented with 10% fetal bovine serum (Corning) at 37 °C under 5% CO2.

### Immunohistochemistry (IHC) analysis

IHC was performed on paraffin-embedded human CRC tissue sections according to standard LSAB protocols (Dako), using primary antibodies against GLYR1 (Proteintech, USA), MLH1 (Proteintech, USA), and Ki-67 (ZSGB-BIO). The degree of staining in the sections was observed and scored independently by three pathologists. According to the criteria based on related references [24, 25], the percentage of GLYR1 and MLH1 positivity was scored from 0 to 5 as follows: 0, < 5%; 1, 5–25%; 2, 25–50%, 3, 50–75%, 4, 75–95% and 5, > 95%. The staining intensity was scored according to a 4-point scale as follows: 0 (no staining); 1 (weak staining, light yellow); 2 (moderate staining, yellowish brown); and 3 (strong staining, brown). Subsequently, GLYR1 and MLH1 expression was calculated by multiplication of the percentage positivity score and staining intensity score, resulting in a final score ranging from 0 to 15. Accordingly, the final expression level of GLYR1 and MLH1 was defined as low (0–4), moderate (5–9) and high (10–15). Ki-67 was positively located in the nucleus and appeared as brownish-yellow staining. For the Ki-67 proliferation index, the average number of positively stained cells as a percentage of the total cells was calculated in 10 randomly selected high-power fields (HPFs).

### Western blot analysis

Total proteins were isolated from cells using lysis buffer (FDbio) and the concentration was determined using BCA protein assay kits (FDbio). Total proteins were separated by 10% or 12% sodium dodecyl sulfate-polyacrylamide gel electrophoresis (SDS-PAGE) and transferred to polyvinylidene fluoride (PVDF) membrane. The membranes were blocked with 5% skimmed milk for 1 h at room temperature and then immunoblotted with primary antibodies at 4 °C overnight. Subsequently, the membranes were incubated with goat anti-rabbit or anti-mouse secondary antibody (ZSGB-BIO) and visualized by enhanced chemiluminescence. Details of the primary antibodies are shown in Supplementary Table S1.

### GLYR1 exon13 mutation detection

CRC cells were cultured normally. When the cells were grown in good condition, cell pellets were collected and temporarily stored at − 80 °C prior to use. Paraffin-embedded human CRC tissues were cut into sections (10 μm thick) and were immediately sent to Guangzhou BioTruthSearch Co., Ltd. for Sanger sequencing. GLYR1 exon13 mutations detected in the CRC cell lines are shown in Table S2.

### Small interfering RNA (siRNA) and short hairpin RNA (shRNA) transfection

The siRNA sequences were as follows: GLYR1-Homo-464 (5′-GGAAAGAAGAGGGUGUCUUTT-3′, siGLYR1–1), GLYR1-Homo-262 (5′-GGGUAAACGAUUCCAGCAATT-3′, siGLYR1–2) and were purchased from GenePharma (Shanghai, China). GLYR1-Homo-464 (5′-GGAAAGAAGAGGGUGUCUUTT-3′) was selected to construct an interfering plasmid carrying the hU6-MCS-CMV-GFP-SV40-neomycin vector (shGLYR1) provided by Genechem (Shanghai, China); a control sequence (5′-TTCTCCGAACGTGTCACGT-3′, shCtrl) was used as a control for shGLYR1. SW480 and SW620 cells were transiently transfected with siRNA or shRNA using Lipofectamine 3000 Transfection Reagent (Invitrogen, USA) according to the manufacturer’s instructions. Stable cell lines were obtained by resistance screening with G418 (Sigma, USA) at a concentration of 800 μg/mL (SW480) or 1000 μg/mL (SW620) for more than 1 month. The G418 resistance screening concentrations were determined in preliminary experiments. GLYR1 transfection efficiency was assessed by quantitative real-time polymerase chain reaction (qRT-PCR) and Western blot analyses.

### RNA extraction and qRT-PCR

Total cellular mRNA was extracted using TRIzol (Invitrogen) and reverse-transcribed into cDNA using PrimeScript RT Reagent Kit with gDNA Eraser (TaKaRa). SYBR Premix Ex Taq (TaKaRa) was used for qRT-PCR. The primers used for qRT-PCR as described in Supplementary Table S3.

### Co-immunoprecipitation (co-IP)

Protein extracts from CRC cells (5 × 10^7^/sample) were immunoprecipitated using the primary antibodies displayed in Supplementary Table S1. Input was used as a positive control and normal rabbit IgG (Cell Signaling Technology) was used as a negative control. The immunoprecipitated proteins were then analyzed by Western blotting.

### Immunofluorescence and co-localization

Cells were washed three times with PBS and fixed with 4% paraformaldehyde (PFA) for 30 min at room temperature. The cells were permeabilized by incubation with 0.25% Triton X-100 (Sigma–Aldrich) for 3–15 min at room temperature and then blocked with goat serum (ZSGB-BIO) for 1 h at 37 °C. The cells were then incubated with the appropriate primary antibodies diluted in PBS at 4 °C overnight. Subsequently, the cells were incubated with the secondary antibody conjugated with fluorescein isothiocyanate (ZSGB-BIO) for 1 h at 37 °C followed by staining with 4′,6-diamidino2-phenylindole (DAPI) at room temperature for 8 min. Images were captured using the FV10-ASW 3.0 Viewer. Details of the primary antibodies used are displayed in Supplementary Table S1.

### Drug treatment

Cell apoptosis was induced by treatment with 5-FU (Sigma) dissolved in DMSO. Cells were seeded in a 6-well plate (2 × 10^5^ cells/well) and incubated for more than 24 h before treatment with 5-FU for 48 h. The concentrations of 5-FU were determined according to the half maximal inhibitory concentration (IC50) obtained in a preliminary experiment. DMSO (0.1%) was used as a vehicle control for 5-FU-treated cells.

Cell differentiation was induced by treatment with all-trans retinoic acid (ATRA; Sigma) dissolved in DMSO. Cells were seeded in a 6-well plate (2000 cells/well) and incubated for more than 24 h before treatment with ATRA for 0, 1, 3, and 5 days (the dosing time was strictly controlled to ensure the total culture time was consistent). The concentrations of ATRA used were determined in a preliminary experiment in which significant morphological changes were observed when SW480 cells were treated with 20 μM ATRA and SW620 cells were treated with 10 μM ATRA for 48 h. DMSO (0.1%) was used as a vehicle control for ATRA-treated cells.

### Flow cytometric analysis

Cell apoptosis and the cell cycle were analyzed by flow cytometry. Cells were cultured in normal medium and transfected with siRNA targeting GLYR1 or the negative control. Cell apoptosis was determined using annexin V-FITC/propidium iodide (PI) apoptosis detection kits (KGA107) according to the manufacturer’s instructions. For cell cycle analysis, cells were fixed in 70% ethyl alcohol for more than 4 h at 4 °C and then analyzed on a flow cytometer after using the cell cycle analysis kit (KGA152) following the manufacturer’s instructions. Experiments were repeated at least three times.

### Hoechst 33258 staining assay

Cell apoptosis was determined by Hoechst 33258 staining. Cells transfected with siRNA targeting GLYR1 or the negative control were treated with 5-FU at the IC50 for 48 h. Apoptotic cells were detected using the Hoechst 33258 staining kit (KeyGEN BioTECH) according to the manufacturer’s instructions and visualized by fluorescence microscopy.

### Cell counting kit-8 (CCK-8) and colony formation assays

Cell viability was tested using CCK-8 and colony formation assays. Cells transfected with shRNA plasmids targeting GLYR1 or a negative control were seeded in a 96-well plate (2000 cells/well) and incubated at 37 °C for 1, 2, 3, and 4 days. Medium containing 10 μL CCK-8 reagent (TransGen Biotech) and 100 μL culture medium was added to each well. After 4 h of incubation, the absorbance at 450 nm was examined on a microplate reader. For colony formation assays, transfected cells were seeded in a 6-well plate (200 cells/well) and cultured for 2 weeks. The cells were then fixed with 4% PFA and stained with Giemsa for 20 min. The number of colonies (> 50 cells/colony) was counted under a light microscope. Experiments were repeated at least three times.

### Statistical analysis

All statistical analyses were performed using the IBM SPSS Statistics 23 and confirmed by statisticians in the Department of Health Statistics, Southern Medical University. Analysis of correlations between GLYR1 expression and histopathological factors was conducted using *χ*^2^ tests and Spearman’s correlation test, and between the expression of GLYR1 and MLH1 using Pearson’s correlation test. Differences between groups were analyzed using a two-tailed paired Student’s t-test. In vitro cell growth assay was determined using Two-way ANOVA. Data were expressed as mean ± standard deviation. *P < 0.05, **P < 0.01 and ***P < 0.001 were considered to indicate statistical significance.

## Results

### GLYR1 is downregulated in MSI CRC

**Fig. 1:**
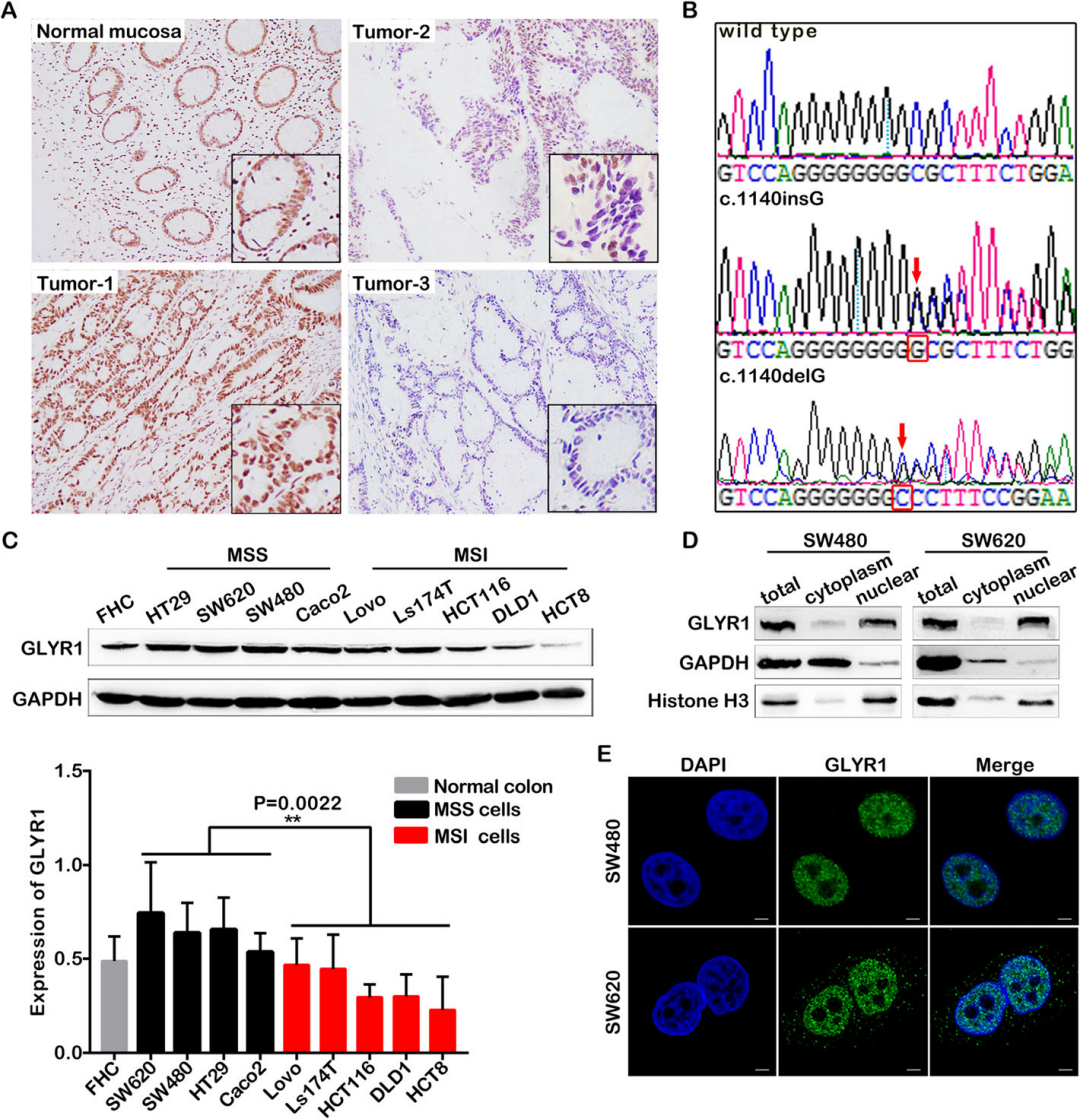
GLYR1 is downregulated in MSI CRC. **a** Representative photographs showing immunohistochemical staining of GLYR1 (× 100, scale = 100 μm) in 221 paraffin-embedded normal human colorectal tissue (normal mucosa) and colorectal cancer tissue (Tumor-1, Tumor-2, Tumor-3) sections. The image in the black pane in the lower right corner of the overlay shows a partial enlargement. **b** GLYR1 exon 13 mutation detection in CRC tissues and cells. Partially intercepted representative GLYR1 exon 13 normal (wild type) and mutant (c.1140insG, c.1140delG) sequences are shown. The red arrowhead indicates the mutation site. **c** Western blot analysis of GLYR1 protein expression in CRC cells. Quantification of protein expression was normalized against GAPDH. Error bars represent the mean ± SD (*n* = 3). Comparison of GLYR1 expression in MSS and MSI cells, **P = 0.0022. **d** Western blot analysis of GLYR1 expression in the cytoplasm and nucleus of SW480 and SW620 cells. **e** Localization of GLYR1 expression in SW480 and SW620 cells by immunofluorescence analysis (× 2400, scale = 5 μm); DAPI (blue) stained nuclei

Immunohistochemistry (IHC) staining was performed in 221 paraffin-embedded CRC tissue sections, including 164 cases of MSS CRC and 57 cases of MSI CRC. As shown in Fig. 1a, differential expression of GLYR1 was observed in these samples. Adjacent normal colorectal tissues (Normal mucosa) and most colorectal tumors (Tumor-1) displayed medium-high nuclear expression, while a small proportion of tumors displayed a partial (Tumor-2) or complete (Tumor-3) deficiency in nuclear expression and this phenomenon was commonly observed in MSI CRC. The correlation between GLYR1 expression level and CRC clinical features was further analyzed (Table 1). The results indicated that GLYR1 expression was closely related to tumor position (*ρ* = − 0.245, *P* = 0.001), tumor size (*ρ* = 0.216, *P* = 0.004), tumor grade (*ρ* = 0.319, *P* < 0.001), mucinous component (*ρ* = − 0.235, *P* = 0.007) and microsatellite instability (*ρ* = − 0.641, *P* < 0.001). According to classification as described in the Methods section, 109 (66.5%) of 164 cases of MSS CRC showed high expression, while 48 (29.3%) cases showed medium expression, and only seven (4.3%) cases showed low expression. In contrast, among the cases of MSI CRC, four (7%) showed high expression, 16 (28.1%) showed medium expression, and 37 (64.9%) showed low expression. We then performed Sanger sequencing to detect GLYR1 exon13 mutations [5] in three paraffin-embedded MSI CRC tissues with GLYR1 expression deficiency and nine CRC cell lines. A single guanine (G) base insertion mutation (c.1140insG) was detected in two of three MSI CRC tissues as well as in four of five MSI CRC cell lines (Ls174T, HCT116, DLD1 and HCT8), while a single base G deletion mutation (c.1140delG) was detected in the fifth cell line (LOVO). However, GLYR1 exon13 mutations were not detected in any of the four MSS CRC cell lines (HT29, SW620, SW480 and Caco2) (Fig. 1b, Table S2). Expression of GLYR1 in the CRC cell lines and the normal human fetal colonic mucosa cell line FHC was evaluated by Western blot analysis. The results confirmed that GLYR1 was downregulated in MSI CRC cell lines compared with MSS CRC cell lines at the protein level (*P* = 0.0022, Fig. 1c).

**Table 1:** The relationship between GLYR1 expression and CRC clinicopathological features

	No.of cases	Low	Medium	High	χ2 value	***P***-value
**Frequency**	221 (100%)	44 (19.9%)	64 (29.0%)	113 (51.1%)		
**Age**						
Younger (< 60 years)	118 (53.4%)	24 (20.3%)	36 (30.5%)	58 (49.2%)	**0.427**	**0.808**
Older (> = 60 years))	103 (46.6%)	20 (19.4%)	28 (27.2%)	55 (53.4%)		
**Gender**						
Male	135 (61.1%)	26 (19.3%)	42 (31.1%)	67 (49.6%)	**0.781**	**0.677**
Female	86 (38.9%)	18 (20.9%)	22 (25.6%)	46 (53.5%)		
**Position**						
Colon	150 (67.9%)	38 (25.3%)	45 (30.0%)	67 (44.7%)	**10.890**	**0.004**
Rectum	71 (32.1%)	6 (8.4%)	19 (26.8%)	46 (64.8%)		
**Tumor sise (maximum diameter)**						
< 5 cm	106 (48.0%)	8 (7.5%)	34 (32.1%)	64 (60.4%)	**19.726**	**< 0.001**
> =5 cm	115 (52.0%)	36 (31.3%)	30 (26.1%)	49 (42.6%)		
**Tumor grade (diffrentiation)**						
G1	27 (12.2%)	1 (4.8%)	6 (28.6%)	14 (66.7%)	**33.570**	**< 0.001**
G2	173 (78.3%)	27 (15.6%)	51 (29.5%)	95 (54.9%)		
G3	21 (9.5%)	16 (59.3%)	7 (25.9%)	4 (14.8%)		
**Invasive depth**						
Submucosal	14 (6.4%)	0 (0.0%)	6 (42.9%)	8 (57.1%)	**9.586**	**0.143**
Myometrium	21 (9.5%)	2 (9.5%)	6 (28.6%)	13 (61.9%)		
Subserosal	92 (41.6%)	16 (17.4%)	26 (28.3%)	50 (54.3%)		
Break the serosa	94 (42.5%)	26 (27.7%)	26 (27.7%)	42 (44.6%)		
**Mucinous component**						
Absent	165 (74.7%)	21 (12.7%)	52 (31.5%)	92 (55.8%)	**21.066**	**< 0.001**
Present	56 (25.3%)	23 (41.1%)	12 (21.4%)	21 (37.5%)		
**Microsatellite instability**						
MSS	164 (74.2%)	7 (4.3%)	48 (29.3%)	109 (66.4%)	**107.823**	**< 0.001**
MSI-L	6 (2.7%)	4 (66.6%)	1 (16.7%)	1 (16.7%)		
MSI-H	51 (23.1%)	33 (64.7%)	15 (29.4%)	3 (5.9%)		
**Lymphatic metastasis**						
Negative	131 (59.3%)	27 (20.6%)	41 (31.3%)	63 (48.1%)	**1.268**	**0.530**
Positive	90 (40.7%)	17 (18.9%)	23 (25.6%)	50 (55.5%)		

Our observation that GLYR1 is downregulated both in MSI CRC tissues and cell lines was further confirmed by bioinformatic analysis using the Oncomine database, *P* = 0.001, fold change in expression = 2 (Supplementary Figure S1A, B). In addition, nucleoplasm separation and immunofluorescence assays confirmed that GLYR1 expression was localized mainly in the nucleus of SW480 and SW620 cells, with no or only weak expression in cytoplasm (Fig. 1d, e).

### GLYR1 interacts with MLH1 and regulates its nuclear expression

**Fig. 2:**
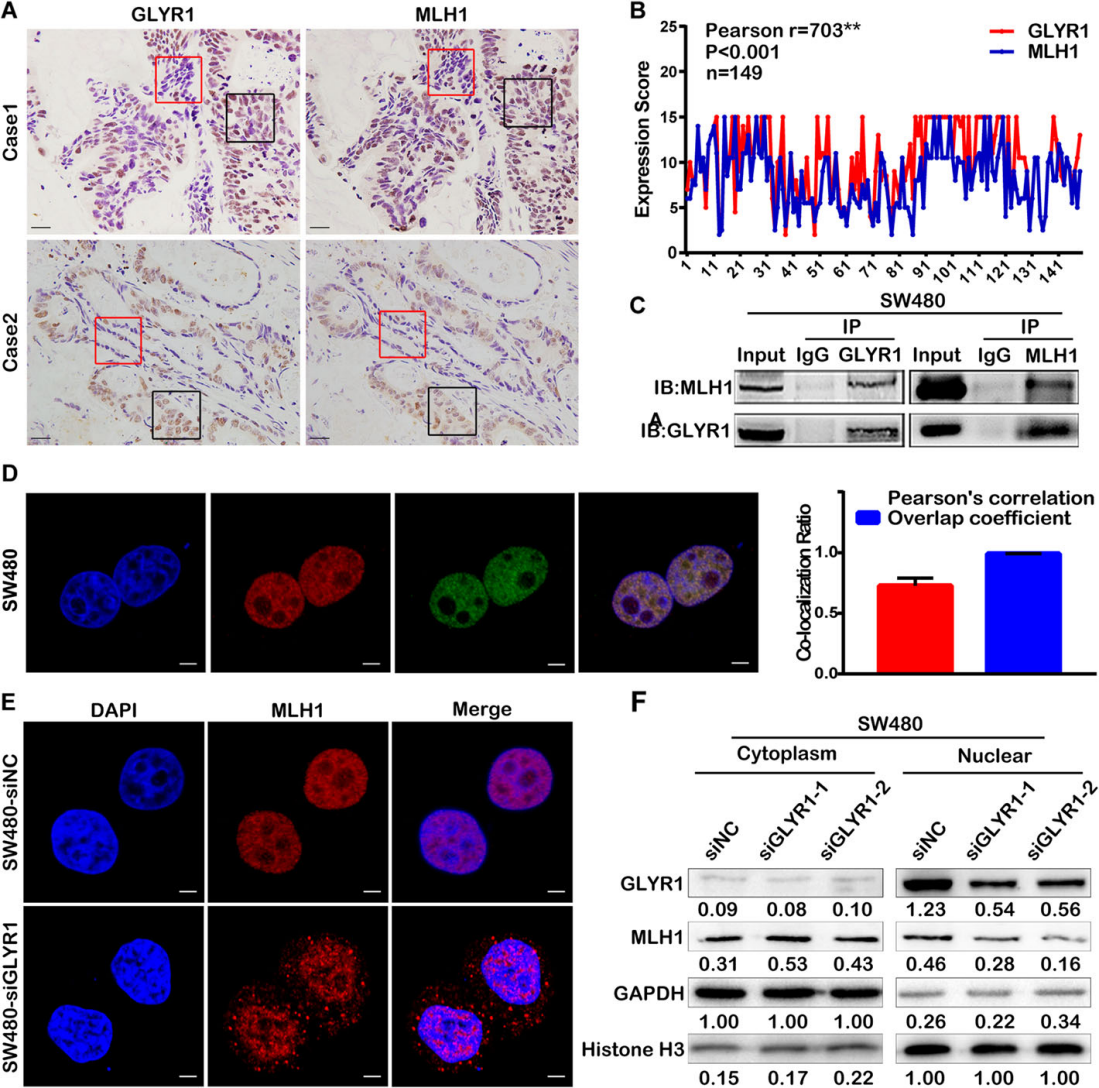
GLYR1 interacts with MLH1 and regulates its nuclear expression. **a-b** Correlation analysis of GLYR1 and MLH1 expression by immunohistochemical staining of paraffin-embedded human colorectal cancer tissue sections. Representative photographs of immunohistochemical staining are shown (200×, scale = 50 μm). The areas in the red and black panes indicate co-deficiency and co-expression, respectively. Pearson’s correlation analysis of GLYR1 and MLH1 according to immunohistochemical staining score, **r = 0.703, ***P < 0.001. **c** Co-immunoprecipitation was performed to validate the interaction between GLYR1 and MLH1 in SW480 cells. **d** Co-localization of GLYR1 (red) and MLH1 (green) in SW480 cells was assessed by laser-scanning confocal microscopy (× 2400, scale = 5 μm), DAPI (blue) stained nuclei. Error bars represent the mean ± SD of the Pearson’s correlation and overlap coefficient of GLYR1 and MLH1 (*n* = 5), *P < 0.05, **P < 0.01, ***P < 0.001. (E-F) Effect of GLYR1 knockdown on the expression and localization of MLH1 analyzed by immunofluorescence (× 2400, scale = 5 μm) and nucleoplasm separation assays. Grayscale values were normalized against GAPDH in the cytoplasm and histone H3 in the nucleus

Mismatch repair deficiency (dMMR) is known to be the most important cause of MSI [26, 27]. To investigate the relationship between GLYR1 and MSI, we analyzed the correlation between GLYR1 and MRR genes using the R2 database [Tumor Colon MSI-status (Core-Transcript) - Sveen - 95 - rma-sketch - huex10t]. A positive correlation was identified between GLYR1 and MLH1, PMS2, MSH2 and MSH6. The correlation coefficients of GLYR1 and MRR genes were 0.408 (*P* < 0.001), 0.468 (*P* < 0.001), 0.339 (*P* < 0.001), and 0.233 (*P* = 0.02), respectively (Supplementary Figure S2A). It has been reported that inactivation of the MLH1 gene through promoter CpG island hypermethylation is the main cause of MSI [28]. Therefore, we investigated the correlation between GLYR1 and MLH1. Immunohistochemical staining of GLYR1 and MLH1 was performed in 149 paraffin-embedded serial CRC tissue sections. As shown in Fig. 2a, GLYR1 expression was consistent with that of MLH1, displaying a co-expression or co-deficiency phenomenon. Pearson correlation analysis showed that among 149 cases of CRC, there were 16 (10.74%) cases with GLYR1 and MLH1 co-expression at low levels, 43 (28.86%) cases with medium expression, and 54 (36.24%) cases with high expression. The correlation coefficient between GLYR1 and MLH1 was 0.703 (*P* < 0.001) (Fig. 2b).

The interaction between GLYR1 and MLH1 in SW480 and SW620 cells was evaluated by co-IP (Fig. 2c, Supplementary Figure S2C). In addition, the co-localization of GLYR1 and MLH1 was validated by immunofluorescence assays. The Pearson’s correlation and overlap coefficients of GLYR1 and MLH1 in SW480, SW620 cells were 0.728 ± 0.064, 0.992 ± 0.0006 (*n* = 5) and 0.675 ± 0.042, 0.991 ± 0.0010 (*n* = 5), respectively (Fig. 2d, Supplementary Figure S2B).

To further explore the regulatory relationship between GLYR1 and MLH1, we used siRNA to knockdown GLYR1 and MLH1 expression. Western blot analysis showed that downregulation of GLYR1 or MLH1 had a little effect on the mutual total protein expression (Supplementary Figure S3A). We then used the protein synthesis inhibitor cycloheximide (CHX) to detect the stability of MLH1 in SW480-siNC and SW480-siGLYR1 cells. As shown in Supplementary Figure S3B, downregulation of GLYR1 slightly decreased the half-life (stability) of MLH1 protein compared with the control cells. These data indicated that GLYR1 downregulation had no significant effect on total protein expression and degradation of MLH1.

Immunofluorescence assays were then performed to analyze the expression and localization of MLH1 in SW480-siNC and SW480-siGLYR1 cells. The results showed that MLH1 was localized mainly in the nucleus, with no or only weak expression in the cytoplasm in SW480-siNC cells. In contrast, cytoplasmic expression of MLH1 increased significantly in SW480-siGLYR1 cells (Fig. 2e). This result was further confirmed by nucleoplasm separation assays. As shown in Fig. 2f, MLH1 expression in the cytoplasmic extract was increased in SW480-siGLYR1 cells compared with that in SW480-siNC cells. In contrast, the expression of MLH1 in the nuclear extract was significantly decreased in SW480-siGLYR1 cells compared with that in SW480-siNC cells.

Collectively, our results showed that downregulation of GLYR1 had no effect on the total protein expression and degradation of MLH1, but decreased its nuclear expression while increasing its cytoplasmic expression. Thus, these observations indicated that GLYR1 controls nuclear expression of MLH1 mainly by regulation of nuclear transport.

### Downregulation of GLYR1 accelerates cell cycle G1/S transition and promotes cell proliferation

In previous studies, the role of GLYR1 in CRC was analyzed by Gene Set Enrichment Analysis (GSEA). KEGG-CELL-CYCLE, KEGG-APOPTOSIS gene sets were positively enriched in the GLYR1 low expression group of CRC samples (Supplementary Figure S1C, D). In the following study, the transient or stable interference cell lines, SW480-siGLYR1/SW480-shGLYR1 and SW620-siGLYR1/ SW620-shGLYR1 were constructed successfully (Supplementary Figure S4A, B). To avoid off-target effects caused by siRNA or shRNA, rescue experiment were performed by expressing an siRNA-resistant form of the gene (Figure S4C), which confirmed that the siRNAs used in this study did not have off-target effects.

**Fig. 3:**
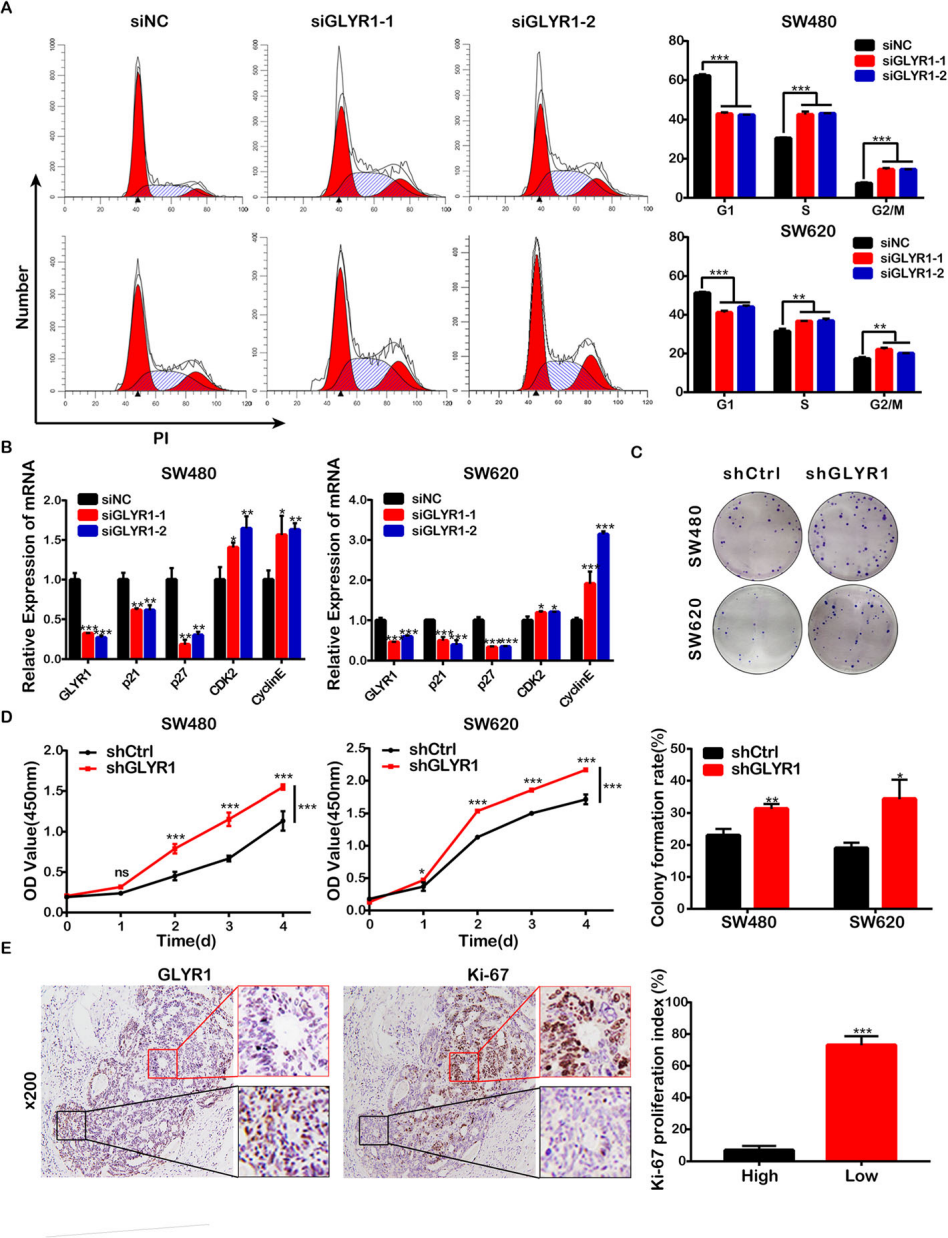
Downregulation of GLYR1 accelerates cell cycle G1/S transition and promotes cell proliferation. **a** Flow cytometric analysis of SW480-siGLYR1 and SW620-siGLYR1 cells after propidium iodide (PI) staining. Error bars represent the mean ± SD of the percentages of cells in the G0/G1, S, and G2/M phases (*n* = 3). **b** Relative mRNA expression of G1/S transition-related genes (p21, p27, CDK2, cyclinE) measured in SW480-siGLYR1 and SW620-siGLYR1 by qRT-PCR; GAPDH was used as an internal control. (**c**-**d**) Cell proliferation was determined by CCK-8 and colony formation assays. Error bars represent the mean ± SD (*n* = 3), *P < 0.05, **P < 0.01, ***P < 0.001. **e** Representative photographs of Ki-67 immunohistochemical staining (× 100, scale = 100 μm) of five paraffin-embedded human CRC tissue sections with partial GLYR1 expression deficiency; the image on the right is a partial enlargement. The proliferation index of Ki-67 was calculated in the regions with intact GLYR1 expression (High) or with deficient GLYR1 expression (Low). Error bars represent the mean ± SD (*n* = 5), *P < 0.05, **P < 0.01, ***P < 0.001

Then, we analyzed the effect of GLYR1 downregulation on the cell cycle progression of SW480 and SW620 cells by flow cytometry. As shown in Fig. 3a, the percentage of siGLYR1 cells in the G0/G1 phase was significantly decreased and accompanied by an increase in both the percentage of cells in the S and G2/M phases compared with siNC cells. These results indicated that downregulation of GLYR1 resulted in accelerated G1/S transition. The cyclinE/CDK2 complex is known to play a vital role in G1/S transition and is negatively regulated by CDK specific inhibitors such as p21 and p27 [29, 30]. Therefore, to further validate our results, the key regulators, p21, p27, cyclin E and cyclin-dependent kinase2 (CDK2) were detected by RT-qPCR. As expected, downregulation of GLYR1 was accompanied by downregulation of the mRNA levels of p21 and p27 compared with the levels detected in siNC cells, while mRNA levels of CDK2 and cyclinE were upregulated (Fig. 3b). These observations further indicated that downregulation of GLYR1 promoted cell cycle progression of CRC cells by accelerating the G1/S transition.

Accelerated cell cycle progression is known to be a common feature of tumor cell proliferation; therefore, we also analyzed the effect of GLYR1 downregulation on CRC cell proliferation in vitro by colony formation and CCK-8 assays. Compared with shCtrl cells, we observed enhanced colony forming and proliferation ability of SW480-shGLYR1 cells (*P* = 0.0046 and *P* < 0.0001, respectively) and SW620-shGLYR1 cells (*P* = 0.0133 and *P* < 0.0001, respectively) (Fig. 3c, d). In addition, we randomly selected five paraffin-embedded human CRC tissue sections with partial GLYR1 expression deficiency for immunohistochemical staining of Ki-67 as a marker of the proliferation index (Fig. 3e). The proliferation index (73% ± 5.701%) was higher in the region with deficient GLYR1 expression (red pane in Fig. 3e). Conversely, the proliferation index (10% ± 2.449%) was lower in the region with intact GLYR1 expression (black pane in Fig. 3e) (*n* = 5, *P* < 0.001). These data revealed that downregulation of GLYR1 accelerated G1/S transition in the cell cycle and promoted cell proliferation of CRC cells in vitro.

### Downregulation of GLYR1 inhibits cell differentiation

**Fig. 4:**
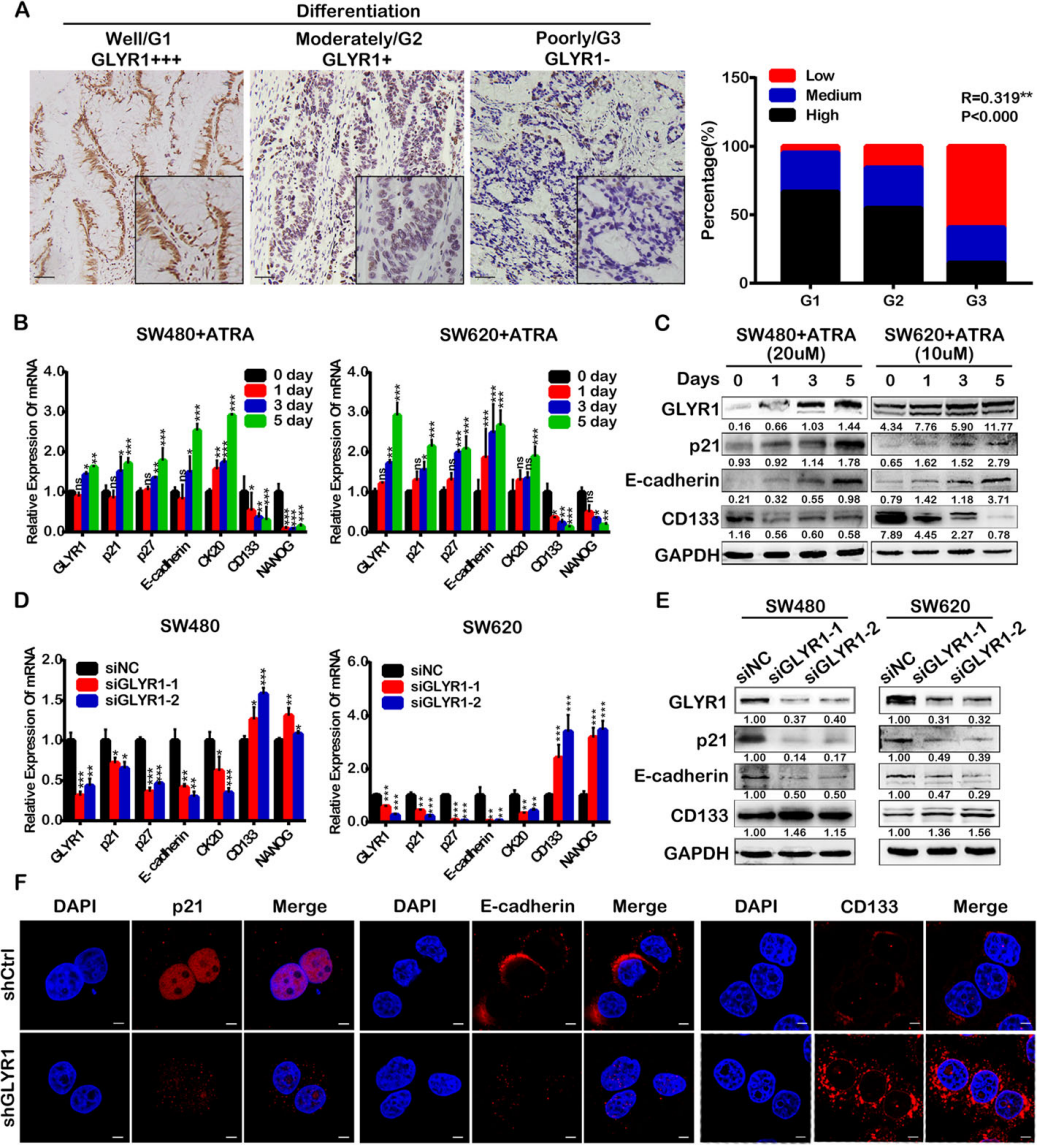
Downregulation of GLYR1 inhibits cell differentiation. **a** Representative photographs of GLYR1 immunohistochemical staining (× 200, scale = 50 μm) in cases with different degrees of differentiation. Spearman’s correlation analysis of GLYR1 expression (Low, Medium, High) and tumor grade (G1, G2, G3),** *R* = 0.319, ***P < 0.001. **b** Relative mRNA expression of cell differentiation-related genes (p21, p27, E-cadherin, CK20, CD133, NANOG) and GLYR1 was measured in SW480 and SW620 cells after ATRA treatment by qRT-PCR; GAPDH was used as an internal control. **c** Western blot analysis of the expression of E-cadherin, CD133, GLYR1, and p21 in SW480 and SW620 cells after ATRA treatment. **d** Relative mRNA expression of cell differentiation-related genes (p21, p27, E-cadherin, CK20, CD133, NANOG) was measured in GLYR1-knockdown SW480 and SW620 cells by qRT-PCR; GAPDH was used as an internal control. **e** Western blot analysis of the expression of E-cadherin, CD133, and p21 in GLYR1-knockdown SW480 and SW620 cells. **f** Immunofluorescent staining of the expression of p21, E-cadherin, and CD133 in GLYR1-knockdown SW480 cells (× 2400, scale = 5 μm); DAPI (blue) stained nuclei. Error bars represent the mean ± SD (*n* = 3), *P < 0.05, **P < 0.01, ***P < 0.001

Previous studies have revealed that GLYR1 expression levels correlate positively with tumor differentiation. As shown in Fig. 4a, high, medium and low GLYR1 expression was detected in 66.7, 28.6, and 4.8% of cases of well-differentiated tumors, respectively; high, medium and low GLYR1 expression was detected in 54.9, 29.5, and 15.6% of cases of moderately-differentiated tumors, respectively; high, medium and low GLYR1 expression was detected in 14.8, 25.9, and 59.3% of cases of poorly-differentiated tumors, respectively (*R* = 0.319, *P* < 0.000). We further validated our results by analyzing the Smith colorectal database (*n* = 177) of Oncomine (Supplementary Figure S5A).

Moreover, at the cellular level, ATRA was used to induce differentiation of CRC cells. As shown in Fig. 4b, expression of the differentiation-associated molecules p21, p27, E-cadherin and CK20 [10, 31–33] was gradually upregulated in SW480 and SW620 cells following ATRA treatment for 0, 1, 3 and 5 days, while expression of stemness-associated molecules CD133 and NANOG [34–36] was downregulated, indicating successful induction of cell differentiation. Cell differentiation was accompanied by simultaneous upregulation of GLYR1 expression (Fig. 4b). The results of Western blot analysis of expression at the protein level were consistent with the results of RT-qPCR analysis (Fig. 4c).

We also knocked down the expression of GLYR1 by siRNA transfection of SW480 and SW620 cells. The results of qRT-PCR and Western blot analyses showed that the expression of p21, p27, E-cadherin and CK20 were downregulated in siGLYR1 cells compared with the levels detected in siNC cells, while CD133 and NANOG were upregulated (Fig. 4d, e). These results were further verified by immunofluorescence assays by detecting the expression of p21, E-cadherin, and CD133 in SW480-shGLYR1 and SW480-shCtrl cells (Fig. 4f). Coincidentally, we found obvious changes in the morphology of shGLYR1 cells. As shown in Supplementary Figure S5B, cells became smaller, spindle-like and with looser connections compared with the morphology of shCtrl cells. Morphological changes in shGLYR1 cells were similar to those observed in epithelial-mesenchymal transition (EMT), which might indicate increased malignancy. These results indicated that downregulation of GLYR1 inhibited CRC cell differentiation, making it a tendency to malignancy.

### Downregulation of GLYR1 decreased CRC cell sensitivity to 5-FU

**Fig. 5:**
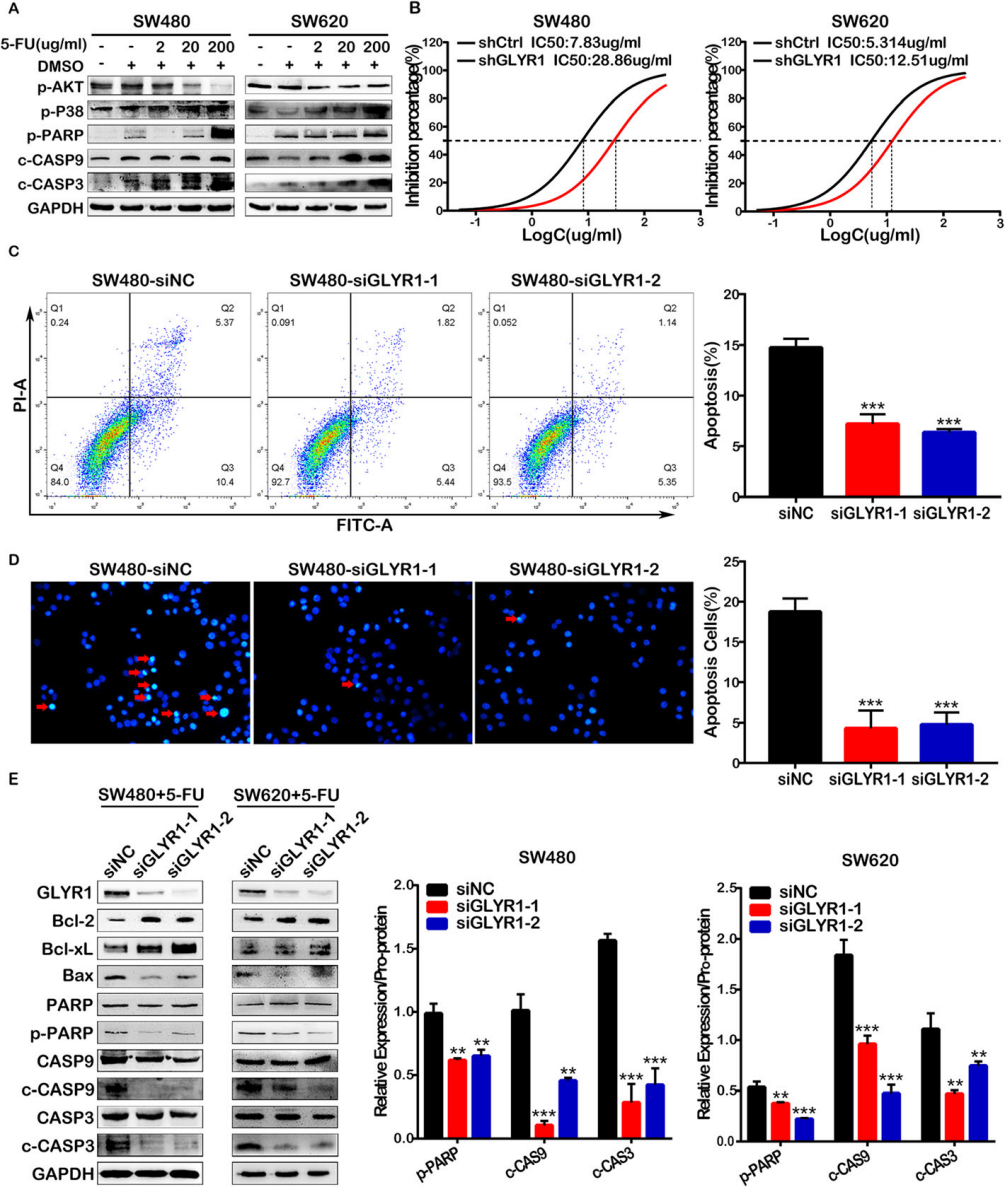
Downregulation of GLYR1 reduces 5-FU-induced apoptosis in CRC cells. **a** 5-FU was used to induce apoptosis in SW480 and SW620 cells and the expression of p-AKT, p-p38, p-PARP, c-CASP9, c-CASP3 was detected by Western blot analysis. **b** CCK-8 assays of GLYR1 downregulation on sensitivity of SW480 and SW620 cells to 5-FU; the half maximal inhibitory concentration (IC50) was calculated using GraphPad software. (**c**-**d**) Effects of GLYR1 downregulation on apoptosis in SW480 cells induced by 5-FU (8.185 μg/ml) treatment for 48 h was determined by flow cytometric analysis and Hoechst 33258 staining. Representative photographs of Hoechst 33258 staining (× 200, scale = 50 μm); red arrowhead indicates positive apoptotic cells. Error bars represent the mean ± SD of apoptosis (n = 3, *n* = 5). **e** Western blot analysis of the expression of the apoptosis-related proteins Bax, PARP, Caspase9, Caspase3 in GLYR1-knockdown SW480 and SW620 cells following 5-FU treatment (8.185 μg/ml, 5.293 μg/ml) for 48 h. Relative expression analysis of cleaved-PARP, cleaved-Caspase9 and cleaved-Caspase3 shown on the right was normalized to the respective pro-protein. Data represent the mean ± SD (*n =* 3), *P < 0.05, **P < 0.01, ***P < 0.001

As the first-line treatment for CRC, 5-FU induces apoptosis of tumor cells, although it has been reported that patients with MSI tumors do not benefit from 5-FU-based chemotherapy [37–39]. However, our GSEA results indicated that GLYR1 is involved in the apoptotic signaling pathway (Supplementary Figure S1D). Thus, we carried out a series of studies to explore the effect of GLYR1 on the sensitivity of CRC cells to 5-FU. First, a gradient concentration of 5-FU (0, 2, 20,200 μg/ml) was used to induce apoptosis of SW480 and SW620 cells. Western blot analysis showed that the expression of p-p38, a key factor in the classical apoptotic pathway [40–42], was upregulated, whereas the expression of p-Akt, a key factor in the anti-apoptotic pathway [43, 44], was downregulated. Moreover, the caspase cascade was activated as shown by the obvious upregulation of the expression of p-PARP, cleaved-caspase9 (c-CASP9), and cleaved-caspase3 (c-CASP3) (Fig. 5a). These results indicated successful induction of apoptosis in SW480 and SW620 cells by 5-FU.

To gain insights into the effect of GLYR1 downregulation on apoptosis in CRC cells, we first performed CCK8 assays and calculated the half maximal inhibitory concentration (IC50) of 5-FU in SW480 (8.185 ± 0.1935 μg/ml) and SW620 (5.293 ± 0.1575 μg/ml) cells (data not shown). The expression of GLYR1 was then knocked down in SW480 and SW620 cells. As shown in Fig. 5b, compared with shCtrl cells, the sensitivity of SW480-shGLYR1 and SW620-shGLYR1 cells to 5-FU was significantly reduced, showing an increase in IC50 from 7.83 ± 0.1625 μg/ml to 28.86 ± 0.1643 μg/ml and from 5.314 ± 0.1547 μg/m to12.51 ± 0.2079 μg/ml, respectively.

Additionally, flow cytometry analysis and Hoechst 33258 staining were performed to validate the effect of GLYR1 downregulation on apoptosis in SW480 (Fig. 5c, d) and SW620 (Supplementary Figure S6A, B) cells. The results showed that GLYR1 downregulation significantly reduced the apoptosis induced in SW40 and SW620 cells by 5-FU compared with that induced in siNC cells. Similarly, at the protein level (Fig. 5e), downregulation of GLYR1 increased the expression of the anti-apoptotic proteins Bcl-2 and Bcl-XL, while the expression of the pro-apoptotic protein Bax [45–47] was decreased, thereby inhibiting the activity of PARP, Caspase9 and Caspase3. Collectively, our results indicated that downregulation of GLYR1 decreased the sensitivity of CRC cells to 5-FU by inhibiting the mitochondrial apoptosis pathway. Therefore, we suggested that downregulation of GLYR1 in MSI CRC might be one of the reasons for its resistance to 5-FU chemotherapy.

### The p38MAPK and PI3K-AKT signaling pathways were involved in the regulation of p21 by GLYR1

**Fig. 6:**
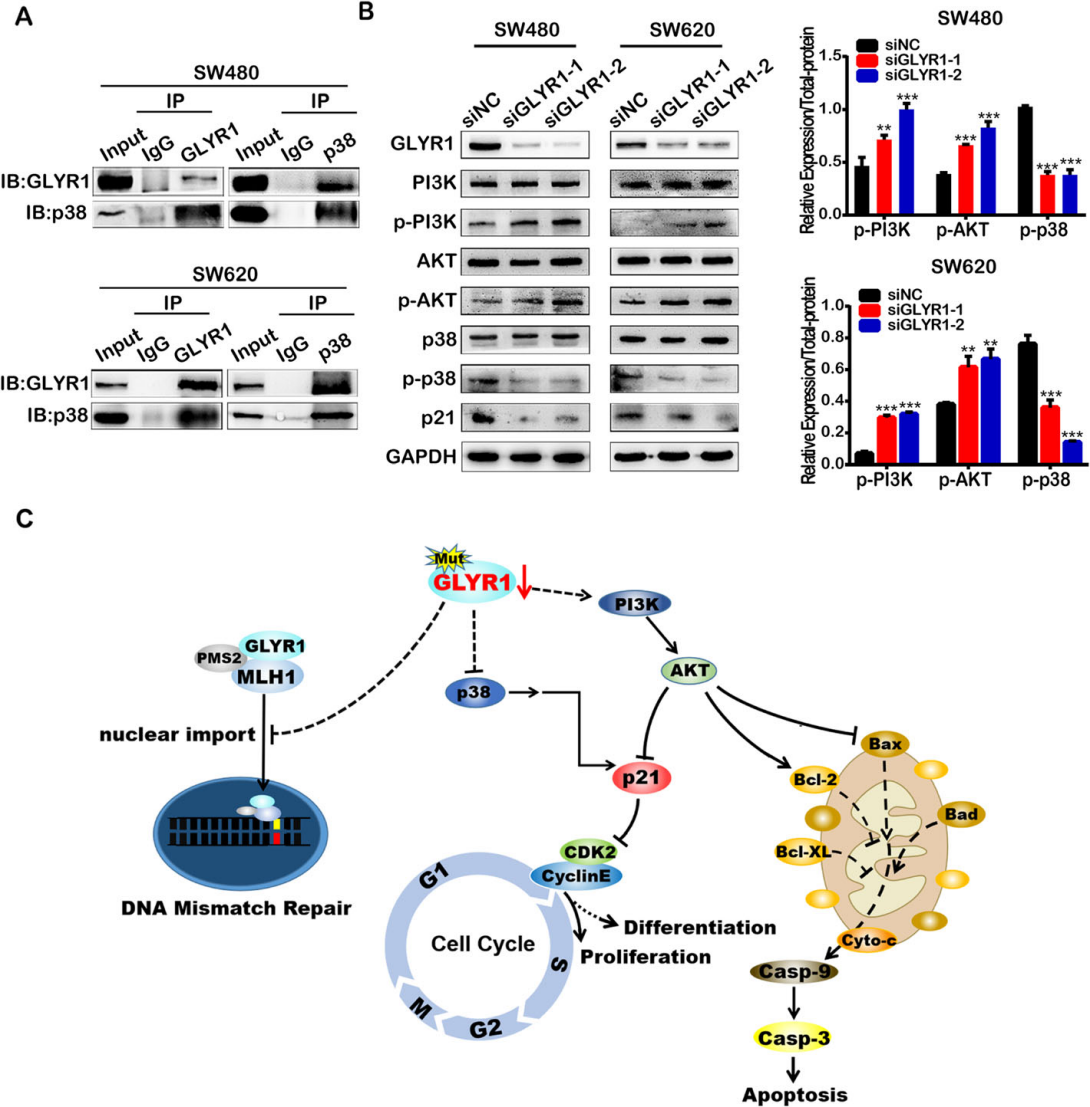
The p38MAPK and PI3K-AKT signaling pathways are involved in the regulation of p21 by GLYR1. **a** Co-immunoprecipitation assays to validate the interaction between GLYR1 and p38 in SW480 and SW620 cells. **b** Western blot analysis of PI3K, p-PI3K, AKT, p-AKT, P38, p-p38, and p21 in SW480-siGLYR1 and SW620-siGLYR1 cells. The expression of p-PI3K and p-AKT was upregulated in the siGLYR1 groups compared with the siNC cells; total protein was used as an internal control. Error bars represent the mean ± SD (*n* = 3), *P < 0.05, **P < 0.01, ***P < 0.001. **c** Molecular mechanism of the function of GLYR1 downregulation in MSI CRC

As previously mentioned, the p38MAPK and PI3K/Akt signaling pathways are the main pathways involved in the regulation of p21, and relevant pathway gene sets were found to be enriched in our GSEA analysis (data not shown). Therefore, we further investigated the effects of GLYR1 downregulation on the key molecules of these two pathways. First, we performed co-IP to validate a previous report of the interaction of GLYR1 with p38 [21] (Fig. 6a). We then further examined the expression of key proteins relayed to the p38MAPK and PI3K/Akt signaling pathways by Western blotting, including p38, p-p38(Thr180/Tyr182), PI3K, p-PI3K (Tyr458/Tyr199), AKT and p-AKT (Ser473). As shown in Fig. 6b, downregulation of GLYR1 decreased p38 phosphorylation but increased the phosphorylation of PI3K and Akt, thereby synergistically downregulating p21 expression. According to the SelTarbase database [48], the SW480 and SW620 cells used in this study were p53 mutants. Therefore, our data indicated that inhibition of p38MAPK and activation of the PI3K-AKT signaling pathway were involved in the decreased p21 expression induced by GLYR1 downregulation, and this effect is mediated via a p53-independent mechanism.

## Discussion

Although a large number of studies have been carried out on MSI CRC, clarification of the molecular events involved in the occurrence and progression of MSI CRC is still urgently required. GLYR1 has a high mutation frequency (51%) in MSI CRC, and has been implicated as a tumor suppressor [5]; however, the role of GLYR1 in tumors is still unclear. Therefore, further investigation of the role of GLYR1 in MSI CRC is of great scientific significance. MLH1, PMS2, MSH2 and MSH6 are the main proteins involved in the DNA mismatch repair (MMR) system. Among them, MLH1-PMS2 (hMutLα) and MSH2-MSH6 (hMutSα) occur in the form of heterodimer [49, 50]. In a recent study, H3K36me3 was shown to recruit hMutSα to chromatin by direct interaction with the PWWP domain of MSH6 [51–53]. However, little is known about the molecular mechanism by which hMutLα is transported to the nucleus, where it subsequently combines with hMutSα. It has been reported that the nuclear import of MutLα is synergistically enhanced by heterodimerization; therefore, redundancy of nuclear localization signals was proposed to ensure the efficient nuclear import of hMutLα [54].

In our previous study, we found a significant correlation between GLYR1 and MSI; therefore, our subsequent findings further indicated that GLYR1 interacts with MLH1 and regulates its nuclear expression. Furthermore, GLYR1 has both a PWWP domain and a nuclear localization sequence (NLS) [21]; therefore, we hypothesized that GLYR1 might synergistically enhance the nuclear import of hMutLα by interaction with MLH1 to regulate its nuclear expression. Our findings highlighted a new possibility for the mechanism of nuclear localization of hMutLα in which downregulation of GLYR1 impairs the function of the MMR system, finally leading to MSI. MSI CRC has the characteristics of large tumor size, low degree of differentiation and insensitivity to 5-FU. Our results demonstrated that downregulation of GLYR1 accelerated the G1/S cell cycle transition, thereby promoting cell proliferation and inhibiting cell differentiation in vitro. Furthermore, GLYR1 downregulation was also found to reduce the sensitivity of CRC cells to 5-FU. However, Fei et al. reported that GLYR1 knockout prolonged the G1 phase and inhibited growth of HeLa cells [55], which was contrary to our results. This discrepancy may be because of the different cell lines used in our study and the function of GLYR1 may be influenced by the heterogeneity of tumors.

Taken together, our results suggested that GLYR1 downregulation is involved in the occurrence and development of MSI CRC. Therefore, further explorations of the involvement of GLYR1 in relevant molecular mechanisms are required.

Since the p38MAPK and PI3K/AKT signaling pathways are known to be involved in the regulation of p21, a preliminary exploration was performed to validate the ability of GLYR1 to regulate p21. As expected, GLYR1 knockdown downregulated p21 by inhibiting p38 phosphorylation while activating the phosphorylation of PI3K and Akt in a p53-independent manner. Due to the high mutation frequency of p53 in most of human cancers, investigation of p53-independent pathways are clearly very important.

GLYR1 has been characterized as a LSD2/KDM1b-specific cofactor that stimulates demethylation of H3K4me1 and H3K4me2. Furthermore, recent studies have shown that GLYR1 functions as a nucleosome-destabilizing factor that facilitates Pol II transcription through nucleosomes. In addition, GLYR1 overexpression was reported to induce p21 transcriptional activation in a p53-independent manner. All of these studies indicated that GLYR1 plays an important role in transcription regulation. Therefore, it can be speculated that transcription is compromised by GLYR1 knockdown. Therefore, genes such as p21 and p27 are likely to be downregulated directly via transcriptional inhibition mediated by GLYR1, although this requires validation in further studies.

## Conclusions

In summary, we found that GLYR1 was downregulated in MSI CRC, which might impair the nuclear import of hMutLα by interacting with MLH1, resulting in disfunction of the MMR system, and finally leading to MSI. In addition, downregulation of GLYR1 in MSI CRC contributed to the typical growth pattern of MSI tumors, including a superior proliferative capacity, poor differentiation and insensitivity to 5-FU. Mechanistically, the p38MAPK and PI3K-AKT signaling pathways were found to be involved in the regulation of p21 by GLYR1 (Fig. 6c). Therefore, our study provides evidence that implicates GLYR1 as a novel target for diagnosis and selection of 5-FU chemotherapy in MSI CRC.

## Supplementary information

**Additional file 1: Figure S1.** GLYR1 is downregulated in MSI CRC and gene sets positively correlated with low GLYR1 expression. Analysis of GLYR1 expression in CRC tissues (A) and CRC cell lines (B) grouped by microsatellites status was performed using Oncomine [56]. (C-D) KEGG-CELL-CYCLE and KEGG-APOPTOSIS gene sets were positive enriched in the low GLYR1 expression group of CRC.

**Additional file 2: Figure S2.** Correlation and interaction between GLYR1 and MLH1. (A) GLYR1 protein expression correlated positively with the MMR genes MLH1, PMS2, MSH2 and MSH6 according to bioinformatics prediction (http://r2.amc.nl). (B) Co-localization of GLYR1 (red) and MLH1 (green) in SW620 cells assessed by laser-scanning confocal microscopy (× 2400, scale = 5 μm); DAPI (blue) stained nuclei. Data represent the mean ± SD of the Pearson’s correlation and overlap coefficients of GLYR1 and MLH1 (*n* = 5), *P < 0.05, **P < 0.01, ***P < 0.001. (C) Co-immunoprecipitation was performed to validate the interaction between GLYR1 and MLH1 in SW620 cells.

**Additional file 3: Figure S3.** Effects of siRNA interference and cycloheximide (CHX) treatment on GLYR1 and MLH1 expression. (A) Western blot analysis of the total protein expression of MLH1 and GLYR1 following siRNA-mediated interference. (B) Western blot analysis of MLH1 protein synthesis in SW480-siNC and SW480-siGLYR1 cells following treatment with the protein synthesis inhibitor cycloheximide (CHX).

**Additional file 4: Figure S4.** Transient or stable interference cell lines were constructed successfully and rescued the siRNA-mediated knockdown of GLYR1 with an optimized gene variant. (A-B) Western blot analysis was performed to verify successful generation of the transient or stable interference cell lines, SW480-siGLYR1/SW480-shGLYR1 and SW620-siGLYR1/ SW620-shGLYR1. (C) SW480 and SW620 cells were transfected with GLYR1 siNC (negative control), or siGLYR1–1/siGLYR1–2 plus the optimized GLYR1 vector (knockdown), or siGLYR1–1/siGLYR1–2 plus the optimized GLYR1 gene (rescue). Western blot analysis of the expression of GLYR1, p-PI3K, p-AKT, p-P38, p21, E-cadherin, CD133, and Bcl-2.

**Additional file 5: Figure S5.** Downregulation of GLYR1 inhibits cell differentiation. (A) Analysis of GLYR1 expression in CRC grouped by tumor grade was performed using Oncomine [56]. (B) Effect of GLYR1 downregulation on the morphology of SW480 and SW620 cells was observed using a general light microscope (× 200, scale = 50 μm).

**Additional file 6: Figure S6.** Downregulation of GLYR1 reduces 5-FU-induced apoptosis in CRC cells. (A-B) Effects of GLYR1 downregulation on apoptosis in SW620 cells following 5-FU (5.293 μg/ml) treatment for 48 h were determined by flow cytometric analysis and Hoechst 33258 staining. Representative photographs of Hoechst 33258 staining (200×); red arrowhead indicates positive apoptotic cells. Error bars represent the mean ± SD of apoptosis (*n* = 3, *n* = 5). *P < 0.05, **P < 0.01, ***P < 0.001.

**Additional file 7: Table S1.** Antibodies used for Western blotting, Coimmunoprecipitation and Immunofluorescence.

**Additional file 8: Table S2.** GLYR1 Exon13 Mutation in CRC cell lines.

**Additional file 9: Table S3.** Primer sequences for qRT-PCR (5′ to 3′).

## Data Availability

All data presented or analyzed in this study are included either in this article or in the additional files.

## Abbreviations

MSI CRC: Microsatellite Instability Colorectal Cancer
GLYR1: Glyoxylate reductase isoform 1
CDK: Cyclin-dependent Kinase
P38MAPK: p38 mitogen-activated protein kinase
MMR: Mismatch repair
NLS: Nuclear localization sequence
