# Correction to: Downregulation of GLYR1 contributes to microsatellite instability colorectal cancer by targeting p21 via the p38MAPK and PI3K/AKT pathways

**DOI:** 10.1186/s13046-020-01635-6

**Published:** 2020-07-06

**Authors:** Zhiyan Hu, Ting Long, Yidan Ma, Jiaxian Zhu, Lingfang Gao, Yan Zhong, Xia Wang, Xiaoyan Wang, Zuguo Li

**Affiliations:** 1 Department of Pathology, Shenzhen Hospital of Southern Medical University, Shenzhen, China; 2 Department of Pathology, Nanfang Hospital, Southern Medical University, Guangzhou, China; 3 Department of Pathology, School of Basic Medical Sciences, Southern Medical University, Guangzhou, China; 4 Guangdong Provincial Key Laboratory of Molecular tumor Pathology, Guangzhou, China

**Correction to: J Exp Clin Cancer Res 39, 76 (2020)**

**https://doi.org/10.1186/s13046-020-01578-y**

Following publication of the original article [1], the authors identified an error in the author author’s affiliation.

The incorrect author’s affiliation is:

Zhiyan Hu^1,2,3†^, Ting Long^2,3†^, Yidan Ma^2,3^, Jiaxian Zhu^2,3^, Lingfang Gao ^2,3^, Yan Zhong ^2,3^, Xia Wang ^1,2,3^, Xiaoyan Wang ^1,2,3^, Zuguo Li ^4,5,6*^

^1^ Department of Pathology, Shenzhen Hospital of Southern Medical University, Shenzhen, China.

^2^ Department of Pathology, Nanfang Hospital, Southern Medical University, Guangzhou, China.

^3^ Department of Pathology, School of Basic Medical Sciences, Southern Medical University, Guangzhou, China.

^4^ Guangdong Provincial Key Laboratory of Molecular tumor Pathology, Guangzhou, China.

^5^ Department of Pathology, School of Basic Medical Sciences, Southern Medical University,

^6^ Guangdong Provincial Key Laboratory of Molecular tumor Pathology, Guangzhou, China.

The correct author’s affiliation is:

Zhiyan Hu^2,3,4†^, Ting Long^3,4†^, Yidan Ma^3,4^, Jiaxian Zhu^3,4^, Lingfang Gao^3,4^, Yan Zhong^3,4^, Xia Wang^2,3,4^, Xiaoyan Wang^2,3,4^ and Zuguo Li^1,3,4*^

^1^ Department of Pathology, Shenzhen Hospital of Southern Medical University, Shenzhen, China.

^2^ Department of Pathology, Nanfang Hospital, Southern Medical University, Guangzhou, China.

^3^ Department of Pathology, School of Basic Medical Sciences, Southern Medical University, Guangzhou, China.

^4^ Guangdong Provincial Key Laboratory of Molecular tumor Pathology, Guangzhou, China.

The author group has been updated above and the original article [1] has been corrected.
