# Supplementary figures and images for: Downregulation of GLYR1 contributes to microsatellite instability colorectal cancer by targeting p21 via the p38MAPK and PI3K/AKT pathways
Source: J Exp Clin Cancer Res. 2020 May 5;39:76. doi: 10.1186/s13046-020-01578-y (PMC7201645; doi:10.1186/s13046-020-01578-y)

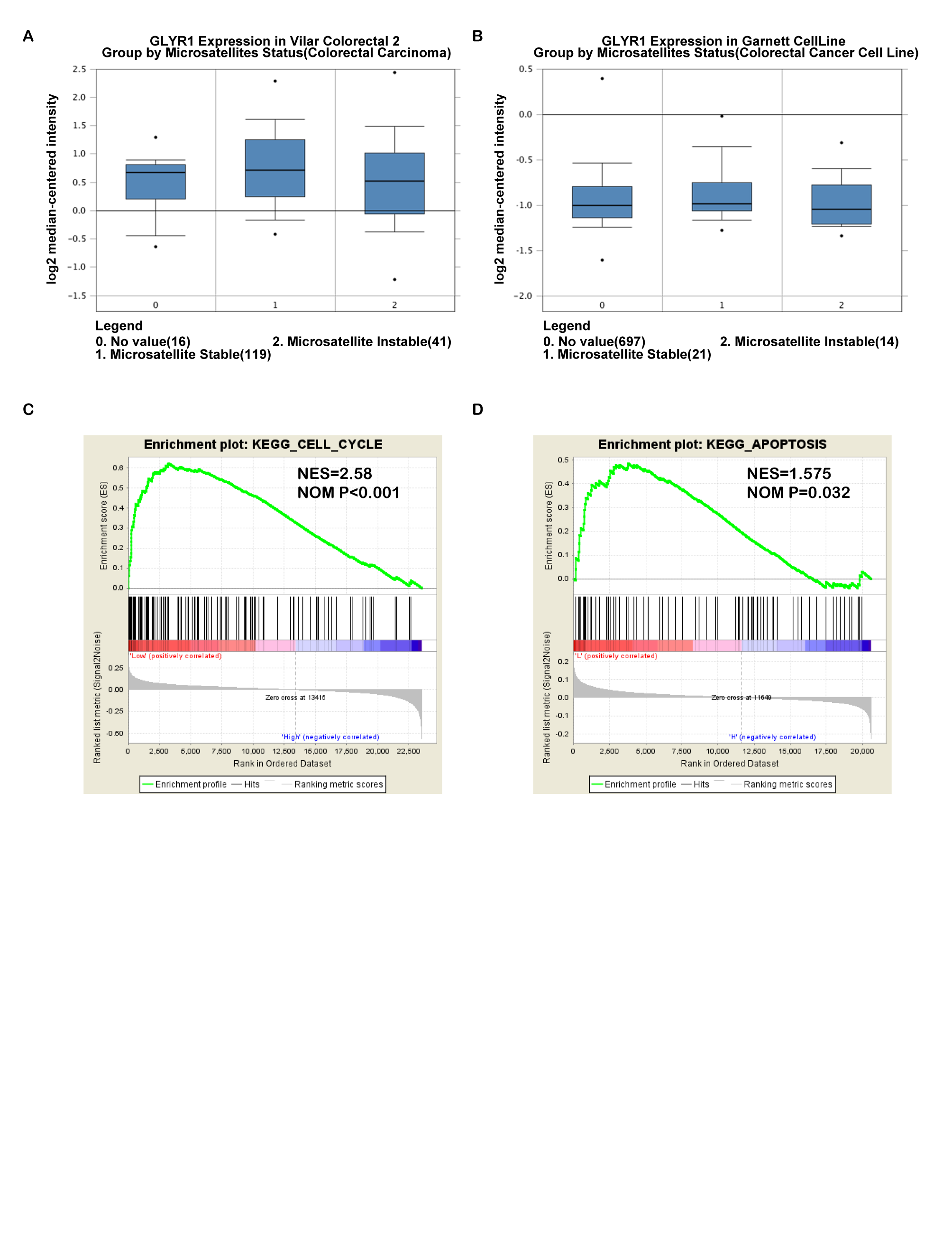

Supplement: Supplementary file 1 — Additional file 1: Figure S1. GLYR1 is downregulated in MSI CRC and gene sets positively correlated with low GLYR1 expression. Analysis of GLYR1 expression in CRC tissues (A) and CRC cell lines (B) grouped by microsatellites status was performed using Oncomine [56]. (C-D) KEGG-CELL-CYCLE and KEGG-APOPTOSIS gene sets were positive enriched in the low GLYR1 expression group of CRC. [file 13046_2020_1578_MOESM1_ESM.tif]

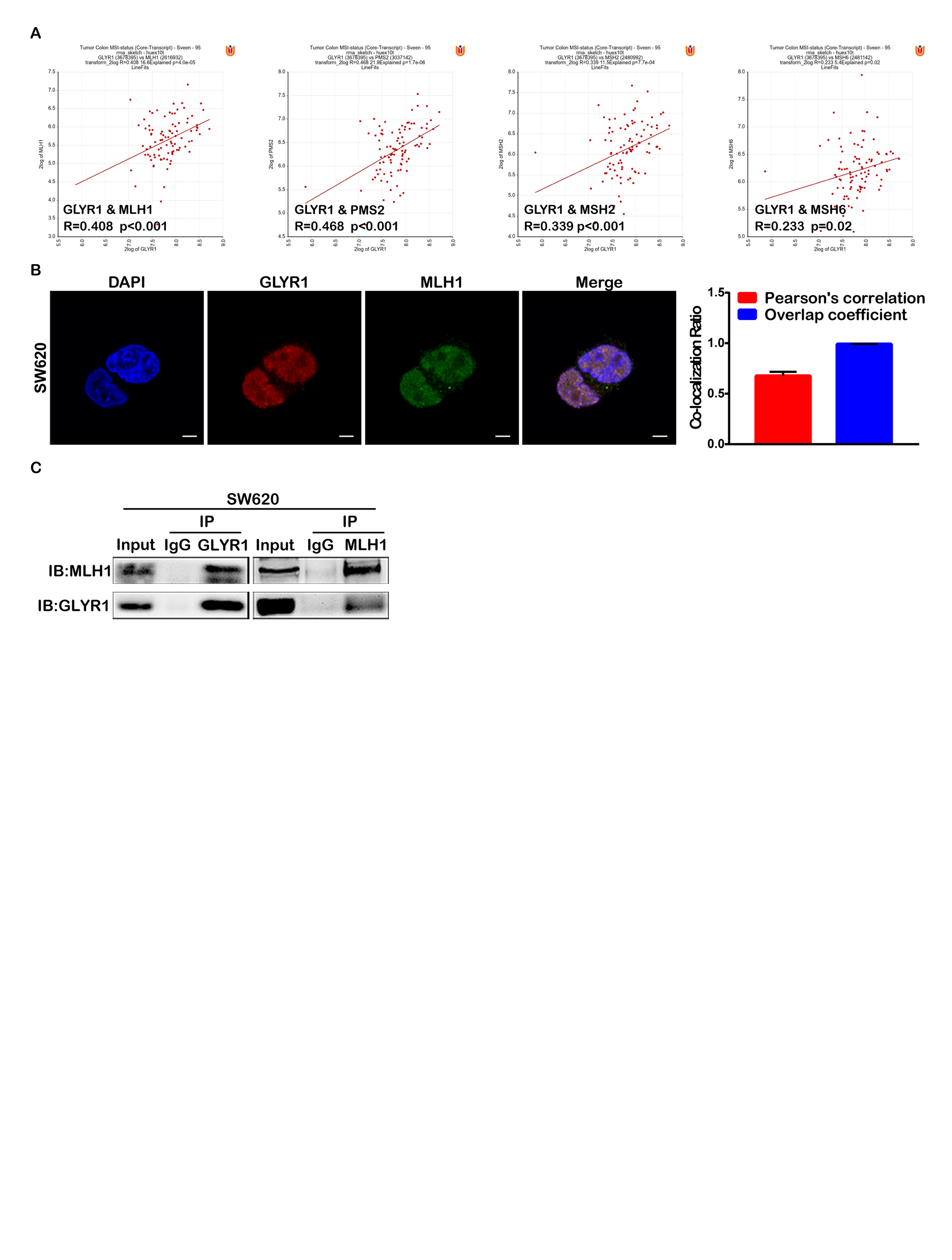

Supplement: Supplementary file 2 — Additional file 2: Figure S2. Correlation and interaction between GLYR1 and MLH1. (A) GLYR1 protein expression correlated positively with the MMR genes MLH1, PMS2, MSH2 and MSH6 according to bioinformatics prediction (http://r2.amc.nl). (B) Co-localization of GLYR1 (red) and MLH1 (green) in SW620 cells assessed by laser-scanning confocal microscopy (× 2400, scale = 5 μm); DAPI (blue) stained nuclei. Data represent the mean ± SD of the Pearson’s correlation and overlap coefficients of GLYR1 and MLH1 (n = 5), *P < 0.05, **P < 0.01, ***P < 0.001. (C) Co-immunoprecipitation was performed to validate the interaction between GLYR1 and MLH1 in SW620 cells. [file 13046_2020_1578_MOESM2_ESM.tif]

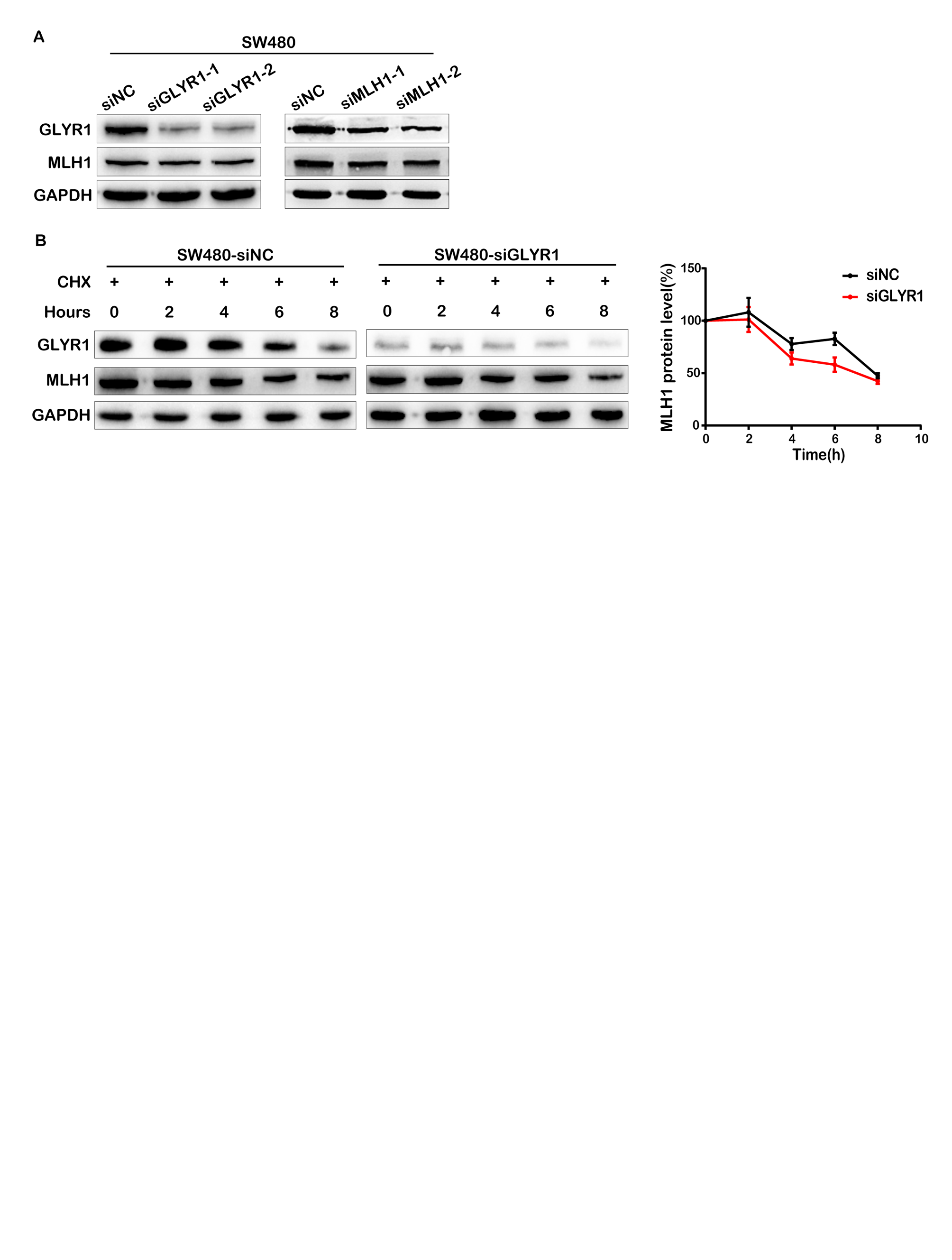

Supplement: Supplementary file 3 — Additional file 3: Figure S3. Effects of siRNA interference and cycloheximide (CHX) treatment on GLYR1 and MLH1 expression. (A) Western blot analysis of the total protein expression of MLH1 and GLYR1 following siRNA-mediated interference. (B) Western blot analysis of MLH1 protein synthesis in SW480-siNC and SW480-siGLYR1 cells following treatment with the protein synthesis inhibitor cycloheximide (CHX). [file 13046_2020_1578_MOESM3_ESM.tif]

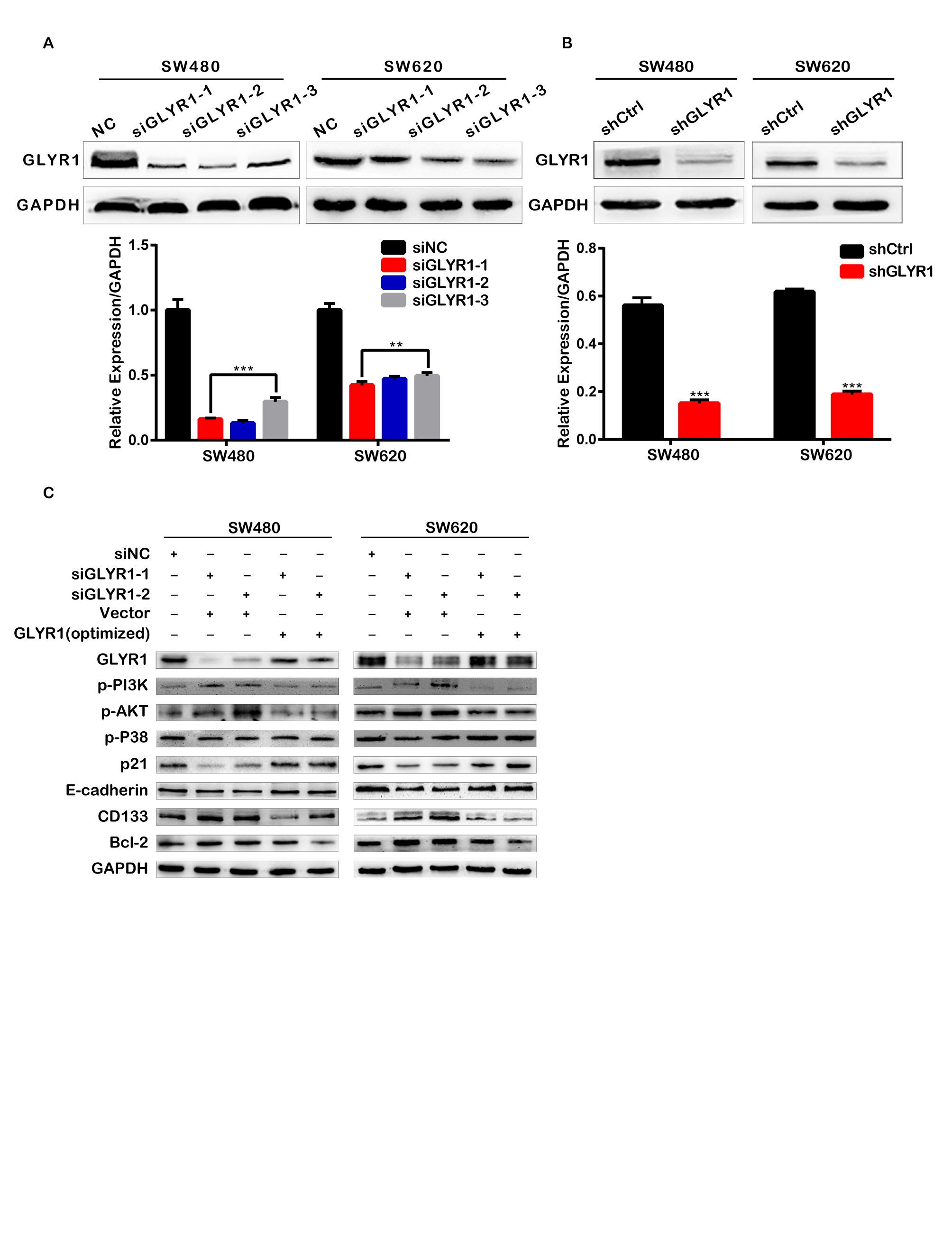

Supplement: Supplementary file 4 — Additional file 4: Figure S4. Transient or stable interference cell lines were constructed successfully and rescued the siRNA-mediated knockdown of GLYR1 with an optimized gene variant. (A-B) Western blot analysis was performed to verify successful generation of the transient or stable interference cell lines, SW480-siGLYR1/SW480-shGLYR1 and SW620-siGLYR1/ SW620-shGLYR1. (C) SW480 and SW620 cells were transfected with GLYR1 siNC (negative control), or siGLYR1–1/siGLYR1–2 plus the optimized GLYR1 vector (knockdown), or siGLYR1–1/siGLYR1–2 plus the optimized GLYR1 gene (rescue). Western blot analysis of the expression of GLYR1, p-PI3K, p-AKT, p-P38, p21, E-cadherin, CD133, and Bcl-2. [file 13046_2020_1578_MOESM4_ESM.tif]

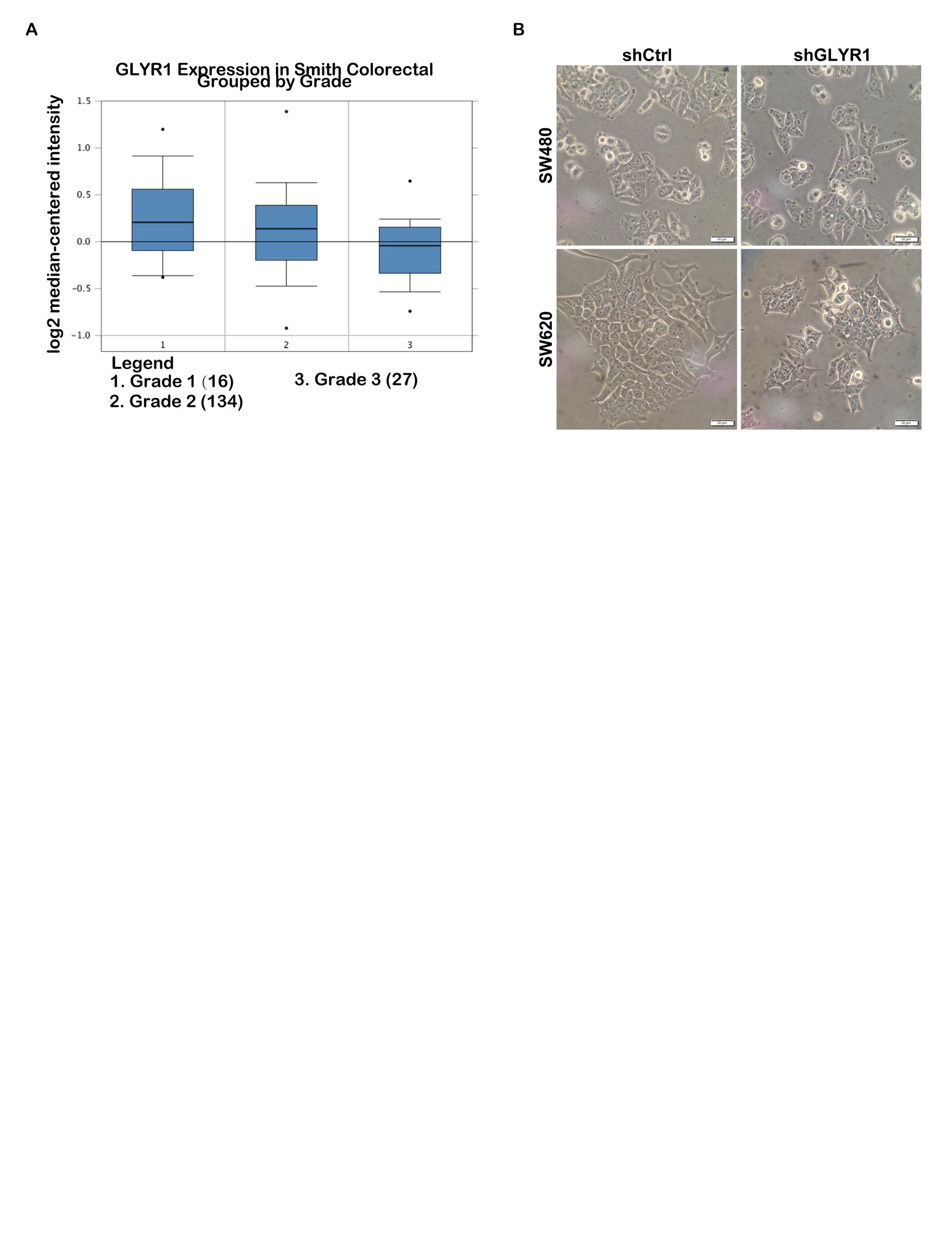

Supplement: Supplementary file 5 — Additional file 5: Figure S5. Downregulation of GLYR1 inhibits cell differentiation. (A) Analysis of GLYR1 expression in CRC grouped by tumor grade was performed using Oncomine [56]. (B) Effect of GLYR1 downregulation on the morphology of SW480 and SW620 cells was observed using a general light microscope (× 200, scale = 50 μm). [file 13046_2020_1578_MOESM5_ESM.tif]

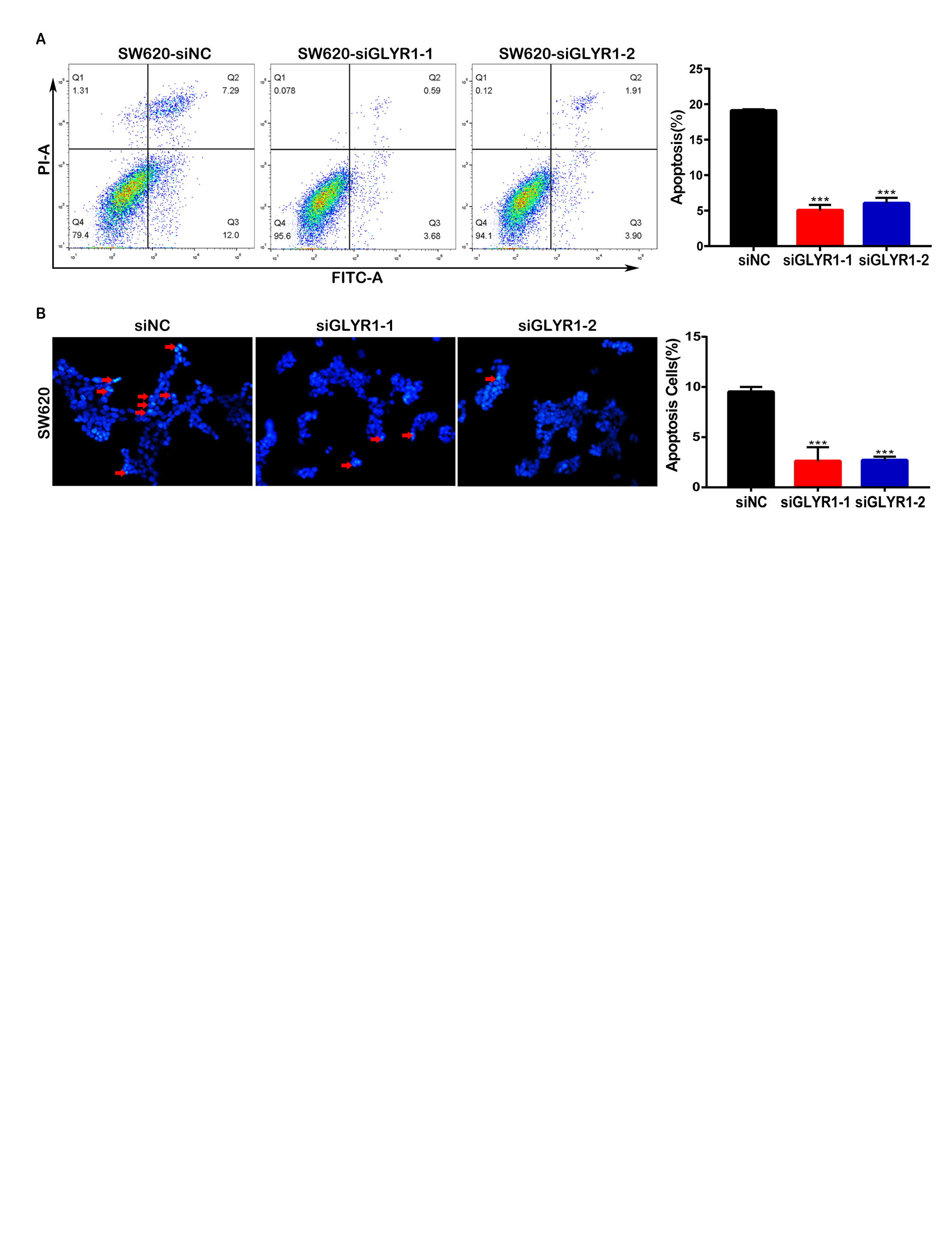

Supplement: Supplementary file 6 — Additional file 6: Figure S6. Downregulation of GLYR1 reduces 5-FU-induced apoptosis in CRC cells. (A-B) Effects of GLYR1 downregulation on apoptosis in SW620 cells following 5-FU (5.293 μg/ml) treatment for 48 h were determined by flow cytometric analysis and Hoechst 33258 staining. Representative photographs of Hoechst 33258 staining (200×); red arrowhead indicates positive apoptotic cells. Error bars represent the mean ± SD of apoptosis (n = 3, n = 5). *P < 0.05, **P < 0.01, ***P < 0.001. [file 13046_2020_1578_MOESM6_ESM.tif]
