# Supplementary material for: Downregulation of GLYR1 contributes to microsatellite instability colorectal cancer by targeting p21 via the p38MAPK and PI3K/AKT pathways
Source: J Exp Clin Cancer Res. 2020 May 5;39:76. doi: 10.1186/s13046-020-01578-y (PMC7201645; doi:10.1186/s13046-020-01578-y)
Supplement: Supplementary file 7 — Additional file 7: Table S1. Antibodies used for Western blotting, Coimmunoprecipitation and Immunofluorescence. [file 13046_2020_1578_MOESM7_ESM.docx]

**Table S2** Antibodies used for Western blotting, Coimmunoprecipitation and Immunofluorescence

| **Antibody** | **Manufacturer** | **Country** | **dilution** |
| --- | --- | --- | --- |
| **Western blotting** | | |  |
| GLYR1 | Proteintech | USA | 1:500 |
| Bcl-2 | Cell Signalling Technology | USA | 1:1000 |
| Bax | Cell Signalling Technology | USA | 1:1000 |
| Bcl-xL | Cell Signalling Technology | USA | 1:500 |
| PARP | Cell Signalling Technology | USA | 1:500 |
| Pro-Caspase9 | Cell Signalling Technology | USA | 1:1000 |
| Cleaved-Caspase9 | Cell Signalling Technology | UK | 1:500 |
| Caspase3 | Proteintech | USA | 1:500 |
| p38 | Proteintech | USA | 1:600 |
| p-p38 | ABclonal | CHN | 1:600 |
| PI3K | Cell Signalling Technology | UK | 1:1000 |
| p-PI3K | Cell Signalling Technology | USA | 1:1000 |
| Akt | Cell Signalling Technology | UK | 1:1000 |
| p-Akt | Cell Signalling Technology | USA | 1:1000 |
| p21 | Proteintech | USA | 1:600 |
| CD133 | ABclonal | CHN | 1:1000 |
| E-cadherin | Cell Signalling Technology | USA | 1:500 |
| GAPDH | Proteintech | USA | 1:2000 |
| **Coimmunoprecipitation** | | |  |
| GLYR1 | Proteintech | USA | 2ug/ml |
| MLH1 | Proteintech | USA | 2ug/ml |
| p38 | ABclonal | CHN | 2ug/ml |
| IgG | Cell Signalling Technology | USA | 2ug/ml |
| **Immunofluorescence** | | |  |
| GLYR1 | Proteintech | USA | 1:100 |
| GLYR1 | SANTA CRUZ | USA | 1:50 |
| MLH1 | Proteintech | USA | 1:100 |
| p21 | Abcam | UK | 1:200 |
| p-p38 | ABclonal | CHN | 1:100 |
| CD133 | ABclonal | CHN | 1:100 |
| E-cadherin | Proteintech | USA | 1:200 |
