# Supplementary material for: Downregulation of GLYR1 contributes to microsatellite instability colorectal cancer by targeting p21 via the p38MAPK and PI3K/AKT pathways
Source: J Exp Clin Cancer Res. 2020 May 5;39:76. doi: 10.1186/s13046-020-01578-y (PMC7201645; doi:10.1186/s13046-020-01578-y)
Supplement: Supplementary file 8 — Additional file 8: Table S2. GLYR1 Exon13 Mutation in CRC cell lines. [file 13046_2020_1578_MOESM8_ESM.docx]

**Table S1** GLYR1 Exon13 Mutation in CRC cell lines

| **Cell** | **MRR** | **GLYR1 Exon13 Mutation** |
| --- | --- | --- |
| SW620 | MSS | Wide-type |
| SW480 | MSS | Wide-type |
| HT29 | MSS | Wide-type |
| Caco2 | MSS | Wide-type |
| LOVO | MSI-H | c.1140delG |
| Ls174T | MSI-H | c.1140insG |
| HCT116 | MSI-H | c.1140insG |
| DLD1 | MSI-H | c.1140insG |
| HCT8 | MSI-H | c.1140insG |
