# Supplementary material for: Downregulation of GLYR1 contributes to microsatellite instability colorectal cancer by targeting p21 via the p38MAPK and PI3K/AKT pathways
Source: J Exp Clin Cancer Res. 2020 May 5;39:76. doi: 10.1186/s13046-020-01578-y (PMC7201645; doi:10.1186/s13046-020-01578-y)
Supplement: Supplementary file 9 — Additional file 9: Table S3. Primer sequences for qRT-PCR (5′ to 3′). [file 13046_2020_1578_MOESM9_ESM.docx]

**Table S3** Primer sequences for qRT-PCR (5' to 3')

| **Gene** | **Forward primer（5' - 3'）** | **Reverse primer（5' - 3'）** |
| --- | --- | --- |
| GLYR1 | AGCGCAAACTTAGCCTGTCTG | TCCGGGATGGTGAGATCCTTC |
| p21 | CTTTGCTGAGATGGTGACTCG | GGAGAGGGTGAGATTAGGGC |
| p27 | AGTGTCTAACGGGAGCCCTA | CTTTGGGTCCACCAAATGCG |
| CDK2 | CTTTGCTGAGATGGTGACTCG | GGAGAGGGTGAGATTAGGGC |
| CyclinE | ATGATTATGAAGCTGTTGGATCTCT | CTGCTCTGCTTCTTACCGCT |
| E-cadherin | AAAGGCCCATTTCCTAAAAACCT | TGCGTTCTCTATCCAGAGGCT |
| CK20 | TCAGTACAGTGGGCATGCAG | GCGTTGGTTTCGTACCACTG |
| CD133 | TTCTTGACCGACTGAGACCCA | TCATGTTCTCCAACGCCTCTT |
| NANOG | TTTGGGATTGGGAGGCTTTG | TCATGTCATTACGATGCAGCAA |
| GAPDH | GGAGCGAGATCCCTCCAA AAT | GGCTGTTGTCATACTTCTCATGG |
